# Serum lead, mercury, manganese, and copper and DNA methylation age among adults in Detroit, Michigan

**DOI:** 10.1093/eep/dvac018

**Published:** 2022-09-21

**Authors:** Evans K Lodge, Radhika Dhingra, Chantel L Martin, Rebecca C Fry, Alexandra J White, Cavin K Ward-Caviness, Agaz H Wani, Monica Uddin, Derek E Wildman, Sandro Galea, Allison E Aiello

**Affiliations:** Department of Epidemiology, Gillings School of Global Public Health, University of North Carolina at Chapel Hill, 135 Dauer Dr, Chapel Hill, NC 27599, USA; Carolina Population Center, University of North Carolina at Chapel Hill, 123 W Franklin St, Chapel Hill, NC 27516, USA; School of Medicine, University of North Carolina at Chapel Hill, 321 S Columbia St, Chapel Hill, NC 27599, USA; Department of Environmental Sciences and Engineering, Gillings School of Global Public Health, University of North Carolina at Chapel Hill, 135 Dauer Dr, Chapel Hill, NC 27599, USA; Institute for Environmental Health Solutions, University of North Carolina at Chapel Hill, 135 Dauer Dr, Chapel Hill, NC 27599, USA; Department of Epidemiology, Gillings School of Global Public Health, University of North Carolina at Chapel Hill, 135 Dauer Dr, Chapel Hill, NC 27599, USA; Carolina Population Center, University of North Carolina at Chapel Hill, 123 W Franklin St, Chapel Hill, NC 27516, USA; Center for Environmental Health & Susceptibility, University of North Carolina at Chapel Hill, 135 Dauer Dr, Chapel Hill, NC 27599, USA; Department of Environmental Sciences and Engineering, Gillings School of Global Public Health, University of North Carolina at Chapel Hill, 135 Dauer Dr, Chapel Hill, NC 27599, USA; Center for Environmental Health & Susceptibility, University of North Carolina at Chapel Hill, 135 Dauer Dr, Chapel Hill, NC 27599, USA; Epidemiology Branch, National Institute of Environmental Health Sciences, A323 David P Rall Building, Research Triangle Park, NC 27709, USA; Center for Public Health and Environmental Assessment, US Environmental Protection Agency, 104 Mason Farm Rd, Chapel Hill, NC 27514, USA; Genomics Program, College of Public Health, University of South Florida, 12901 Bruce B Downs Blvd, Tampa, FL 33612, USA; Genomics Program, College of Public Health, University of South Florida, 12901 Bruce B Downs Blvd, Tampa, FL 33612, USA; Genomics Program, College of Public Health, University of South Florida, 12901 Bruce B Downs Blvd, Tampa, FL 33612, USA; School of Public Health, Boston University, 715 Albany St, Boston, MA 02118, USA; Department of Epidemiology, Gillings School of Global Public Health, University of North Carolina at Chapel Hill, 135 Dauer Dr, Chapel Hill, NC 27599, USA; Carolina Population Center, University of North Carolina at Chapel Hill, 123 W Franklin St, Chapel Hill, NC 27516, USA

**Keywords:** lead, mercury, manganese, copper, DNA methylation, epigenetic age

## Abstract

Although the effects of lead, mercury, manganese, and copper on individual disease processes are well understood, estimating the health effects of long-term exposure to these metals at the low concentrations often observed in the general population is difficult. In addition, the health effects of joint exposure to multiple metals are difficult to estimate. Biological aging refers to the integrative progression of multiple physiologic and molecular changes that make individuals more at risk of disease. Biomarkers of biological aging may be useful to estimate the population-level effects of metal exposure prior to the development of disease in the population. We used data from 290 participants in the Detroit Neighborhood Health Study to estimate the effect of serum lead, mercury, manganese, and copper on three DNA methylation-based biomarkers of biological aging (Horvath Age, PhenoAge, and GrimAge). We used mixed models and Bayesian kernel machine regression and controlled for participant sex, race, ethnicity, cigarette use, income, educational attainment, and block group poverty. We observed consistently positive estimates of the effects between lead and GrimAge acceleration and mercury and PhenoAge acceleration. In contrast, we observed consistently negative associations between manganese and PhenoAge acceleration and mercury and Horvath Age acceleration. We also observed curvilinear relationships between copper and both PhenoAge and GrimAge acceleration. Increasing total exposure to the observed mixture of metals was associated with increased PhenoAge and GrimAge acceleration and decreased Horvath Age acceleration. These findings indicate that an increase in serum lead or mercury from the 25th to 75th percentile is associated with a ∼0.25-year increase in two epigenetic markers of all-cause mortality in a population of adults in Detroit, Michigan. While few of the findings were statistically significant, their consistency and novelty warrant interest.

## Background

The health effects of low-concentration and long-term exposure to lead (Pb) and mercury (Hg) are poorly understood, although the effects of high-dose exposure are well known [1]. Pb, for example, is a well-known toxic metal that can cause neurological damage, cognitive delays, anemia, cardiovascular disease, immunological dysfunction, and more [1-3]. Although the Centers for Disease Control and Prevention currently defines an “elevated blood lead level” as a venous blood specimen with a Pb concentration ≥3.5 µg/dL [4], it is widely accepted that Pb can induce adverse health effects at any level [2]. The same is true for Hg, known to induce neurological, kidney, and gastrointestinal disease at almost any concentration [1, 5]. Biologically essential metals such as manganese (Mn) and copper (Cu) can also induce disease at high exposure levels sometimes found in industrial settings or in heavily contaminated urban areas, although they are less frequently the focus of environmental epidemiologic research [6-8]. Biological aging is an integrative progression of the molecular and physiologic changes that make one more at risk for disease and mortality, unlike distinct disease states induced by toxic exposures [9]. Given that Pb, Hg, and other metals are associated with a diverse array of abnormal physiologic conditions, these metals may induce broad impacts on the aging process in addition to their narrow impacts on distinct acute or chronic diseases. However, estimating the biological age of an organism is a difficult process that has historically depended on the measurement of chromosomal changes (telomere length [10], etc.) or the manifestation of multiple chronic diseases [9]. Epigenetic clocks are highly accurate predictors of mortality risk and chronic disease and have proven to be more predictive of physiologic decline than other biological aging measures [11, 12]. Epigenetic clocks, thus, may be useful when estimating the population-level health effects of exposure to toxins like Pb and Hg [13, 14].

Epigenetic clocks leverage the presence of DNA methylation (DNAm) at cytosine–phosphate–guanine (CpG) dinucleotides within a tissue sample to generate an estimate of the age of the tissue donor [11]. This approach is somewhat analogous to older tools like telomere length, which captured the aging process by measuring the length of telomere “caps” on the ends of chromosomes [9]. Although DNA methylation is a normal biological process integral to the regulation of gene transcription and cellular activity [15], the development of epigenetic clocks such as those by Horvath [16], Levine [17], and Lu [18] highlights the ability of DNA methylation to correlate with the aging process in a regular and predictable manner across populations. The epigenetic clocks derived from DNAm measures are also robustly predictive of health and mortality across the lifespan, as individuals with an estimated biological age greater than their chronological age (termed “age acceleration”) are at increased risk for disease and death [16-19]. There is robust evidence that toxic environmental exposures such as Pb [20-23] and Hg [24, 25] have diverse impacts on the epigenome [2, 13, 14]. Estimating the effects of Pb and Hg on epigenetic aging provides an opportunity to understand their impacts on biological aging and later-life mortality prior to the observance of chronic disease and mortality in the population, which can take decades to manifest.

Here, we estimated the individual and joint effects of serum Pb, Hg, Mn, and Cu on DNAm age using longitudinal data collected from a representative cohort of adults living in Detroit, Michigan, from 2008 to 2013 in the Detroit Neighborhood Health Study (DNHS). Unlike Pb and Hg, which are toxic at almost any concentration, Mn and Cu are biologically essential metals at the proper dose [6, 8] but can cause adverse health effects at exceedingly high or low levels [7, 26, 27]. These four metals, then, may provide insight into the effects of elevated exposure to metal toxins and abnormally high or low exposure to essential trace metal elements on epigenetic aging in a population living in an environmentally contaminated post-industrial city in the Midwestern USA.

## Methods

### Study Population

The DNHS was a longitudinal cohort study of adults in Detroit, Michigan, conducted in five annual waves from 2008 to 2013. The DNHS was designed to recruit a representative sample of the Detroit population by random invitation of households within a random selection of block groups within each of Detroit’s 54 historic neighborhoods. One adult from each selected household was invited to participate in the study, and 53.0% of selected adults enrolled [28]. Recruitment occurred in both Waves 1 (2008–09, *n = *1547 newly recruited participants) and 2 (2009–10, *n *= 534 newly recruited participants), with a total of *n *= 2081 unique participants completing at least one wave of the study. The DNHS Wave 1 sample was representative of the Detroit population in age, sex, race, income, and educational attainment [28, 29]. Following recruitment in Wave 1 or 2, participants were reinvited to participate every year until study completion in Wave 5 (2012–13). Participants completed an annual 40-minute structured telephone interview on their sociodemographic and health history during each wave of the DNHS and could elect to provide venous blood specimens in Waves 1, 2, 4, and 5 (see Supplementary Table S1 for the proportion of participants providing venous blood in each wave). Participation in Wave 5 was limited *a priori* to participants who had provided a biospecimen in any previous wave. Previous research has demonstrated that participants who provided biospecimens were comparable to all participants in Wave 1 [29].

### Metals Assessment

Whole blood samples were collected in trace metal-free tubes during Waves 1, 2, 4, and 5 and were centrifuged and clotted to produce serum for long-term storage at −80°C. Prior to measurement, serum samples were heated in an 80°C hot water bath for 18 hours after adding an equal volume of 70% trace metal grade nitric acid (Fisher). After heating, 30% hydrogen peroxide (Supelco) was added to each sample to digest any remaining organic matter. All sample digestion occurred in DigiTubes (SCPScience, Quebec, CA) to prevent external trace metal contamination. Each sample was diluted with pico-pure water to a final nitric acid concentration of 2–5% after sample digestion. The concentration of Pb, Hg, Mn, and Cu in each serum sample was measured by inductively coupled plasma mass spectrometry (ICP-MS) using a NexION 300D ICP-MS (Perkin Elmer, USA) with SC4-DX autosamplers (ESI, USA). About 2% of nitric acid was used as a carrier and rinse solution. A 1.5 mL sample loop was used to increase sample throughput. We used standard ICP-MS operating procedures to measure ^208^Pb, ^55^Mn, and ^63^Cu. We used helium gas for the measurement of ^202^Hg to prevent interference by argon and carbon. All ICP-MS parameters were optimized as defined by the manufacturer (see Supplementary Table S2). We used TraceCERT® (Sigma–Aldrich, USA) to create seven-point calibration curves for each metal. We used Seronorm® (Accurate Chemical and Scientific Corporation) as a referent. We measured five replicates for each sample. We replaced final concentrations below the limit of detection (LOD) with each metal’s LOD divided by $\sqrt 2 $ (see Supplementary Table S3 for each LOD and the percentage of samples below the LOD). Final serum metal concentrations were natural-log transformed to approximate a normal distribution (Supplementary Table S4) as ln(µg/L). Serum assessment of Pb and Hg is thought to capture long-term exposure (e.g. months to years), as both accumulate in biological tissue and continuously shift between the body’s tissues and the cellular and acellular blood [5, 30]. Mn and Cu, both biologically crucial trace elements, are continuously ingested in many common foods and excreted through the feces [6, 8]. Serum Mn captures long-term exposure due to Mn accumulation in bone (similar to Pb) [6]. Cu accumulation and toxicity only occurs in rare instances, and serum Cu measures short-term exposures only [8].

### DNA Methylation and Methylation Age Assessment

Funding for DNAm measurement was provided by an NIH grant (R01 MD0117728) focused on epigenetic variability among African Americans, resulting in an epigenetic sample that over-represents non-Hispanic African Americans in our analytic cohort. Whole blood samples collected in Waves 1, 2, 4, and 5 were stored at −80°C. Peripheral blood leukocyte-derived DNA was bisulfite converted using the EZ-96 DNA methylation kit (Zymo Research, USA). Bisulfite converted samples were then profiled using either the Illumina Infinium HumanMethylation450K BeadChip (termed “450K”) or the Illumina Infinium MethylationEPIC BeadChip (termed “850K”) according to standard manufacturer protocols (Illumina Inc., USA). Samples were randomized and uniformly distributed across factors responsible for potential batch effects (e.g. participant, sex) to ensure an equal distribution of samples on each plate. Although both Illumina 450K and 850K platforms were used, the epigenetic biomarkers are robust to platform differences, and we included an adjustment for the array to further correct for array-specific (design and/or processing) effects, as described later in the Methods. The resulting DNAm data were subjected to quality control (QC) procedures in preparation for analysis. QC in the 450K dataset included removal of samples with call rates below 90% and low-expressed samples (i.e. mean signal intensity less than half of the 450K dataset-wide median or <2000 arbitrary units) using the *CpGAssoc* R package [31]. Probes with detection *P*-values <0.001 were removed and probes for which ≥10% were missing were filtered out. Cross-hybridizing probes and probes associated with known single-nucleotide polymorphisms (SNPs) were also removed [32]. Of 225 samples processed from the 450K platform, two technical replicates were removed, and five (2.2%) did not pass QC procedures.

In the 850K data, QC metrics from the BeadArray Controls Reporter Software Guide were evaluated for each sample using the *ewastools* R package, and samples failing on any metric were removed [33]. These QC metrics are the same as the standard Illumina processing provided in GenomeStudio®. Sex was estimated from DNAm using the *minfi* R package [34], and sex-discordant samples (samples with disagreement between reported and DNAm-estimated sex) were removed. As in the 450K dataset, samples with call rates below 90% and low-expressed samples were removed using the *CpGAssoc* R package [31]. Probes with detection *P*-values <0.01 were removed and probes for which ≥10% were missing were filtered out. SNPs for each sample were examined for expected distributions of genotypes (i.e. two homozygous genotypes and one heterozygous genotype), and those samples with outlying distributions were excluded. Cross-hybridizing probes and probes associated with known SNPs were also removed [35]. Of 500 samples processed from the 850K platform, 43 (8.6%) did not pass QC procedures.

For both 450K and 850K DNAm data, missing DNAm values were imputed using the *k*-nearest neighbor method, and beta values were converted to *M*-values in advance of correction for sources of technical variation. Batch effects associated with chip or chip position were removed using the ComBat function in the *sva* R package [36]. After the ComBat correction, *M*-values were converted back to beta values, and imputed values were converted back to missing values. The 450K and 850K data were then separately subset to the required input loci for Horvath’s DNA methylation age calculator (https://dnamage.genetics.ucla.edu/).

Background-corrected methylation beta values without normalization were uploaded post-QC to Horvath’s calculator to estimate DNAm age scores developed by Horvath (Horvath Age) [16], Levine *et al.* (PhenoAge) [17], and Lu *et al.* (GrimAge) [18]. We selected the “normalization” option on the online calculator. Horvath Age was developed using data from the Illumina 27K and 450K platforms [16], but it is missing 17 loci when measured on the 850K platform [37]. An updated imputation process (termed “New Methylation Age Calculator” on https://dnamage.genetics.ucla.edu/) improves absolute DNAm age estimates due to the missing loci on the 850K platform, and the Horvath clock remains an accurate predictor of DNAm age in midlife when measured using 850K data [37]. Horvath Age is highly correlated with chronological age in multiple tissue types [16] but is a poorer marker of physiologic dysfunction than PhenoAge and GrimAge [11, 19]. PhenoAge and GrimAge were developed using CpGs present on both the Ilumina 450K and 850K platforms in combination with other blood-based biomarkers [17, 18], and both are robustly predictive of physiologic decline and all-cause mortality [11, 17-19]. All three metrics of DNAm age are strongly correlated with chronological age (Supplementary Fig. S1) and each other (Supplementary Fig. S2) in our analytic cohort. Although all three clocks were primarily developed using data from white Americans and/or Europeans, the initial validation of PhenoAge and GrimAge included Black/African American populations, and each clock consistently performs well in Black populations [17, 18, 38]. For this analysis, DNAm age residuals for each epigenetic clock (Horvath Age, PhenoAge, and GrimAge) were calculated by regressing each DNAm age variable on each participant’s chronological age at the time of venous blood collection. The resulting positive and negative residuals are used as the outcome of interest in our models, with positive regression residuals interpreted as accelerated DNAm aging in this population. Regression residuals were calculated separately for 450K and 850K data.

### Statistical Analysis

Beginning with a sample of 5672 DNHS surveys from 2081 unique participants completed over five waves, we sequentially removed all surveys completed in Wave 3 (when no biospecimens were collected), all samples without serum metals data, and all samples without DNAm data. After applying these exclusion criteria (Supplementary Fig. S3), our analytic cohort included 290 unique DNHS participants with 497 unique survey responses. About 151 participants had only one survey response, 84 had two responses, 42 had three responses, and 13 had four responses. We summarized participant sociodemographic and health characteristics using means and standard deviations for continuous variables and counts and proportions for categorical variables. We calculated the same summary statistics for the entire DNHS Wave 1 cohort (representative of the Detroit population [28, 29]) for comparison. We used Spearman’s rank correlation coefficient to assess correlations between serum Pb, Hg, Mn, and Cu in the first-collected serum sample for each unique participant in our analysis (*n *= 290).

We employed two different modeling strategies to investigate the effects of Pb, Hg, Mn, and Cu on DNAm age acceleration. The first approach estimated the individual effect of each metal (Pb, Hg, Mn, and Cu) on DNAm age acceleration (Horvath Age, PhenoAge, and GrimAge residuals) using mixed-effects modeling [39] with random intercepts for each participant to control for correlated longitudinal observations. Serum metals were modeled continuously as natural-log transformed variables, and DNAm age was modeled continuously as the regression residuals of DNAm age on chronological age. We used Directed Acyclic Graphs (DAGs) [40] to assess confounders of the relationship between serum metals and epigenetic age (Supplementary Fig. S4), including wave-specific measurements of participant sex (modeled as male versus female), race and ethnicity (modeled as non-Hispanic White, non-Hispanic Black/African American, and other), history of cigarette use (modeled as ever versus never using cigarettes), income (modeled as ≥$25 000 versus <$25 000), educational attainment (modeled as >high school graduate/equivalent versus ≤high school graduate/equivalent), and the percentage of households living at or below the federal poverty limit in a participant’s Census block group of residence (collected from the 2008–12 American Community Survey [41] and modeled continuously). Models are also controlled for the DNAm platform (Illumina 850K versus 450K) to remove potential platform effects.

We conducted two sensitivity analyses. The first evaluated the effect of “high” Pb and Hg (serum levels in the upper tertile) and “abnormal” Mn and Cu (serum levels in the bottom or top 15th percentile) on each DNAm age outcome to evaluate potential non-linear relationships between each metal and DNAm age. We coded the upper tertile of Pb and Hg as “high” because neither metal is biologically essential and both are toxic at any level [3, 5]. We coded the bottom or top 15th percentile of serum Mn or Cu as “abnormal” because both metals are biologically essential but can cause physiologic dysfunction at unusually high or low concentrations [6, 8]. We also tested the upper or lower 10th and 20th percentiles of Mn and Cu to see how sensitive our findings were to different cut-points for these biologically essential metals (see Supplementary Table S5 for the number of participants at each categorical exposure level of Pb, Hg, Mn, and Cu). The second sensitivity analysis re-evaluated all models using only 450K or 850K data to identify any batch effects caused by outcome estimation on both Illumina platforms.

The second modeling approach used Bayesian kernel machine regression (BKMR) with a Gaussian kernel function [42, 43] to model the effect of individual and joint exposure to all four metals on DNAm age acceleration. BKMR is a flexible mixture modeling approach that allows for the estimation and visualization of non-linear and interactive associations between multiple exposure variables and an outcome variable [42]. A detailed computational and theoretical background of BKMR is available elsewhere [42], but the general BKMR model can be depicted as shown in Equation (1), where *Y* represents the outcome for individual *i* at time *j, h()* denotes the exposure–outcome function that allows for non-linear effects and interactions between the components of the exposure mixture (metals *z_1_* to *z_4_*), *b* represents a random intercept for individual *i*, and ***β*** represents the effect of a vector of covariates (***x^’^***) for individual *i* at time *j*.

(1) $${Y_{ij}} = h\left( {{z_{ij1}}, \ldots ,{z_{ij4}}} \right) + {b_i} + {\bf{\it{x}}}_{{\bf{\it{ij}}}}^{\bf{\it{^{\prime}}}}{\beta} + { \in _{ij}}$$

As in our single-metal mixed models, our BKMR models included participant-level random intercepts to account for the correlation among repeated measurements of exposures and outcomes. We controlled for the same set of sociodemographic and health characteristics as in our mixed models and included a fixed term for the Illumina platform (coded as 850K versus 450K) [43]. We ran 50 000 iterations for each BKMR model and included variable selection to report posterior inclusion probabilities (PIPs, interpretable as the importance of an individual mixture component in the model) for each component of the metal mixture. We examined trace plots from each model to confirm model convergence. Based on these BKMR models, we estimated the total effect of the metal mixture on DNAm age by comparing *h()* after setting all metals to a given percentile compared to *h()* when all metals in the mixture were at their 50th percentile. We also plotted the univariate exposure–response function for each metal when all other metals were fixed at the 50th percentile to visualize potential non-linear effects. The effect of joint exposure to two metals was visualized as the exposure–response function for each metal when holding one other metal at the 25th, 50th, or 75th percentile and all remaining metals at the 50th percentile. The effect of individual metals in the mixture was estimated as the effect on *h()* of raising a single metal from its 25th to 75th percentile when all other metals were set to either the 25th, 50th, or 75th percentile.

All tests of statistical significance used *α* = 0.05, and all mapping, data visualization, and analysis were conducted in R (version 4.1.0) [44].

## Results

**Figure 1:**
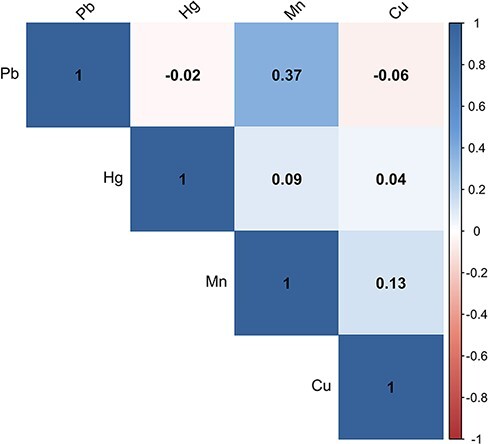
Spearman’s rank correlation coefficient of serum Pb, Hg, Mn, and Cu in 290 samples representing the first serum sample collected from each unique participant

The sociodemographic characteristics of the entire DNHS Wave 1 cohort (representative of the city of Detroit in 2008 [28, 29]) and of Waves 1, 2, 4, and 5 participants included in our analysis are presented in Table 1. Our analytic cohort includes 290 unique DNHS participants providing 497 survey responses and biospecimens. The proportion of participants identified as non-Hispanic African American is higher (88%) in our analytic cohort than in the Detroit-representative Wave 1 cohort (84%). Our cohort is also older and of lower income (57% making <$25 000 versus 42%) than the entire DNHS Wave 1 population, but the distribution of sex, educational attainment, and block group poverty is similar between each group. Our analytic cohort over-represents participants who have ever smoked (73%) compared to the entire Wave 1 cohort (62%). Compared to chronological age, Horvath Age averaged 4.1 years higher (*r *= 0.82), GrimAge averaged 0.2 years lower (*r *= 0.74), and PhenoAge averaged 6.8 years lower (*r *= 0.81) (Supplementary Fig. S1). The untransformed distributions of each serum metal (Supplementary Table S4) are consistent with published reference intervals (<5 µg/L for Hg [45], <2.4 µg/L for Mn [46], and 720–1660 µg/L for Cu [47]), although we are unable to compare our serum Pb distributions with any known serum reference range for that metal. Pairwise Spearman’s rank correlations of each metal (Fig. 1) demonstrated a strong positive correlation between Pb and Mn (*r* = 0.43) and a weaker positive correlation between Mn and Cu (*r* = 0.14). Other pairwise correlations were close to 0.

**Table 1:** Sociodemographic characteristics and mean exposure and outcome levels of DNHS participants included in this analysis. Characteristics of the DNHS Wave 1 cohort (column 2) are provided as a comparison to participants from Waves 1, 2, 4, and 5 included in this analysis (columns 3–6). Column percentages may not sum to 100% due to rounding

		Analytic cohort			
	Detroit-representative cohort (*n *= 1547)	Wave 1 (*n *= 97)	Wave 2 (*n *= 188)	Wave 4 (*n *= 125)	Wave 5 (*n *= 87)
Age (years)					
Mean (SD)	51 (±17)	54 (±14)	55 (±13)	59 (±13)	61 (±14)
Missing	38 (2.5%)	0 (0%)	0 (0%)	0 (0%)	0 (0%)
Sex					
Female	895 (58%)	58 (60%)	116 (62%)	72 (58%)	52 (60%)
Male	652 (42%)	39 (40%)	72 (38%)	53 (42%)	35 (40%)
Race and ethnicity					
Non-Hispanic White	153 (10%)	8 (8%)	23 (12%)	6 (5%)	4 (5%)
Non-Hispanic African American	1295 (84%)	88 (91%)	158 (84%)	116 (93%)	82 (94%)
Other	90 (6%)	1 (1%)	7 (4%)	3 (2%)	1 (1%)
Missing	9 (0.6%)	0 (0%)	0 (0%)	0 (0%)	0 (0%)
Income bracket					
<$25 000	644 (42%)	56 (58%)	107 (57%)	65 (52%)	52 (60%)
≥$25 000	714 (46%)	37 (38%)	76 (40%)	53 (42%)	33 (38%)
Missing	189 (12.2%)	4 (4.1%)	5 (2.7%)	7 (5.6%)	2 (2.3%)
Educational attainment					
≤HS Grad/Equivalent	691 (45%)	50 (52%)	83 (44%)	57 (46%)	38 (44%)
>HS Grad/Equivalent	856 (55%)	47 (48%)	105 (56%)	68 (54%)	49 (56%)
Lifetime smoking					
Never smoker	584 (38%)	21 (22%)	44 (23%)	40 (32%)	24 (28%)
Ever smoker	954 (62%)	76 (78%)	144 (77%)	85 (68%)	63 (72%)
Missing	9 (0.6%)	0 (0%)	0 (0%)	0 (0%)	0 (0%)
Block group poverty, %					
Mean (SD)	36 (±17)	36 (±18)	39 (±18)	36 (±18)	35 (±18)
Missing	53 (3.4%)	0 (0%)	1 (0.5%)	0 (0%)	0 (0%)
Serum ln(Pb)					
Mean (SD)	−1.6 (±1.2)	−1.5 (±1.2)	−1.3 (±1.1)	−1.3 (±0.76)	−1.5 (±1.2)
Missing	1220 (78.9%)	0 (0%)	0 (0%)	0 (0%)	0 (0%)
Serum ln(Hg)					
Mean (SD)	−0.087 (±1.3)	−0.07 (±1.3)	−0.21 (±1.3)	−0.51 (±1.3)	−0.25 (±1.4)
Missing	1234 (79.8%)	3 (3.1%)	4 (2.1%)	4 (3.2%)	0 (0%)
Serum ln(Mn)					
Mean (SD)	0.19 (±0.31)	0.17 (±0.29)	0.46 (±0.77)	0.31 (±0.50)	0.49 (±0.84)
Missing	1220 (78.9%)	0 (0%)	0 (0%)	0 (0%)	0 (0%)
Serum ln(Cu)					
Mean (SD)	7.1 (±0.49)	7.1 (±0.81)	7.1 (±0.50)	7.2 (±0.25)	7.1 (±0.63)
Missing	1220 (78.9%)	0 (0%)	0 (0%)	0 (0%)	0 (0%)
Horvath Age					
Mean (SD)	58 (±10)	58 (±10)	61 (±11)	61 (±9.8)	63 (±9.0)
Missing	1400 (90.5%)	0 (0%)	0 (0%)	0 (0%)	0 (0%)
PhenoAge					
Mean (SD)	47 (±13)	48 (±13)	49 (±13)	50 (±13)	53 (±14)
Missing	1400 (90.5%)	0 (0%)	0 (0%)	0 (0%)	0 (0%)
GrimAge					
Mean (SD)	55 (±15)	54 (±15)	48 (±14)	64 (±11)	66 (±11)
Missing	1400 (90.5%)	0 (0%)	0 (0%)	0 (0%)	0 (0%)

**Figure 2:**
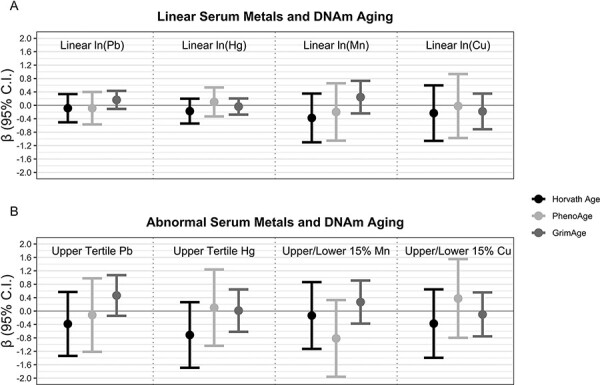
Associations between (A) linear natural-log transformed serum Pb, Hg, Mn, and Cu and (B) upper tertile (versus middle or lower tertile) serum Pb and Hg or upper/lower 15th percentile (versus 16th–84th percentile) serum Mn and Cu and accelerated Horvath Age, PhenoAge, and GrimAge. All models include participant random effects and control for participant sex, race, ethnicity, cigarette use, income, education, block group poverty, and Illumina platform. 95% C.I. = 95% Confidence Interval. Full model results are available in Supplementary Tables 4 and 5

Figure 2 displays mixed effects model results for the estimated effect of serum Pb, Hg, Mn, and Cu on accelerated Horvath Age, PhenoAge, and GrimAge. Pb was positively associated with GrimAge acceleration when exposure was modeled linearly (*β*: 0.16, 95% CI: −0.11, 0.43) or as the upper tertile versus the combined middle and lowest tertiles (*β*: 0.46, 95% CI: −0.14, 1.07), but relationships between Pb and other DNAm metrics were comparatively imprecise and closer to the null of 0 (Supplementary Table S6). Results for Hg clustered close to the null, but Hg in the upper tertile was associated with a 0.71 (95% CI: −1.69, 0.26) year decrease in Horvath Age acceleration compared to Hg in the combined middle or lower tertiles (Supplemental Table S6). Results for Mn and Cu (Supplementary Table S7) were generally less precise and closer to the null, although Mn in the upper or lower 15th percentile was associated with a 0.82 (95% CI: −1.96, 0.33) year decrease in PhenoAge acceleration compared to Mn in the 16th–84th percentiles. The estimated effects of abnormal serum Mn and Cu were extremely sensitive to the cut-point (highest or lowest 10th, or 15th, or 20th percentile) used in our models (Supplementary Table S8). As an example, Cu in the highest or lowest 10th percentile was strongly negatively associated with Horvath Age acceleration (*β*: −1.04, 95% CI: −2.22, 0.14), but Cu in the highest or lowest 20th percentile was not associated with Horvath Age acceleration (*β*: 0.01, 95% CI: −0.96, 0.97). In general, however, “abnormal” Mn (whether the highest or lowest 10th, 15th, or 20th percentile) was consistently negatively associated with PhenoAge acceleration and positively associated with GrimAge acceleration, but all estimates suffered from low precision. Model results when our data were restricted to only those respondents with DNAm measured using the 450K Illumina platform (Supplementary Fig. S5 and Tables S9 and S10) were less precise (with a smaller *n* of 164) and close to the null, while model results from the 850K platform (Supplementary Fig. S6 and Tables S11 and 12) were extremely similar to our overall results.

**Figure 3:**
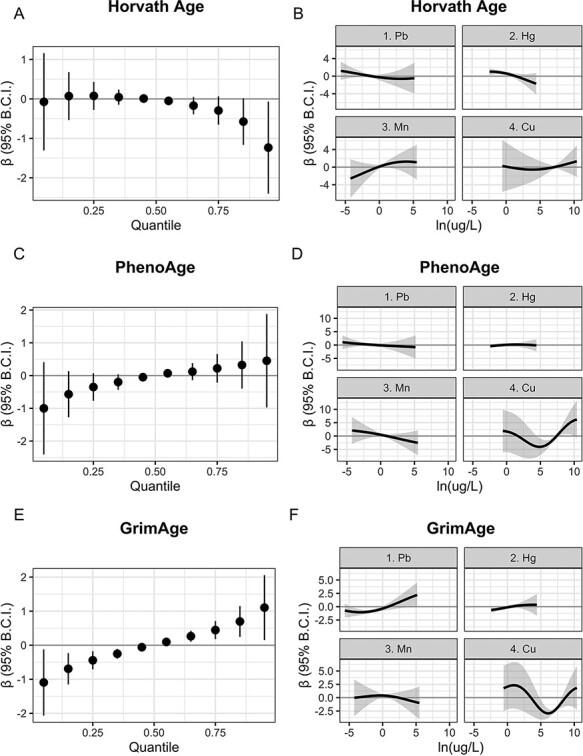
Total effect of metal mixture displayed for accelerated (A) Horvath Age, (C) PhenoAge, and (E) GrimAge. Point estimates and 95% Bayesian credible intervals display estimated effect of joint exposure to all metals at a given quantile compared to the effect of joint exposure to all metals at their respective median. Estimated univariate exposure–response functions and 95% Bayesian credible bands for each metal while holding all other metals constant at the 50th percentile displayed for accelerated (B) Horvath Age, (D) PhenoAge, and (F) GrimAge. All models estimated using BKMR include participant random effects and control for participant sex, race, ethnicity, cigarette use, income, education, block group poverty, and Illumina platform. 95% B.C.I. = Bayesian credible interval

Figure 3 displays the total effect of joint exposure to the metal mixture at a given quantile compared to joint exposure to all metals at their median concentration on accelerated Horvath Age, PhenoAge, and GrimAge (*β* and 95% BCI in Supplementary Table S13). The effect of increasing exposure to the total mixture appeared slightly curvilinear for both Horvath Age acceleration (Fig. 3A) and PhenoAge acceleration (Fig. 3C), with the lowest predicted levels of Horvath Age acceleration at the highest levels of the overall mixture and the lowest levels of PhenoAge acceleration at the lowest levels of the overall mixture. GrimAge acceleration (Fig. 3E), however, steadily increased with increasing exposure to the metal mixture. As an example, joint exposure to all metals at the 75th percentile was associated with a 0.45 (95% BCI: 0.18, 0.71) year increase in GrimAge acceleration, and joint exposure at the 25th percentile was associated with a −0.44 (95% BCI: −0.71, −0.18) year decrease in GrimAge acceleration when compared to joint exposure to all metals at the 50th percentile. Figure 3B, D, and F visualize univariate exposure–response functions for each mixture component and outcome while holding all other metals at the 50th percentile, demonstrating the predominance of non-linear effects in these data. The estimated effect of Pb on Horvath Age acceleration (Fig. 3B) generally clustered near the null with no apparent overall trend, but Horvath Age acceleration decreased with increasing Hg and increased with increasing Mn and Cu. PhenoAge acceleration (Fig. 3D) appeared unassociated with Pb and Hg, decreased with increasing Mn, and demonstrated a strong curvilinear relationship with Cu such that PhenoAge acceleration was positive at the highest and lowest levels of Cu and negative near the median level of Cu. This curvilinear relationship with Cu appeared again with GrimAge acceleration (Fig. 3F). Increasing levels of Pb and Hg were associated with increasing GrimAge acceleration, while the effect of Mn clustered closely to the null. Estimated PIPs for each metal and outcome (Supplementary Table S14) demonstrated that Cu was estimated as the most important mixture component for each DNAm age outcome, followed by Mn and Hg for Horvath Age acceleration, Mn and Pb for PhenoAge acceleration, and Mn and Pb for GrimAge acceleration.

**Figure 4:**
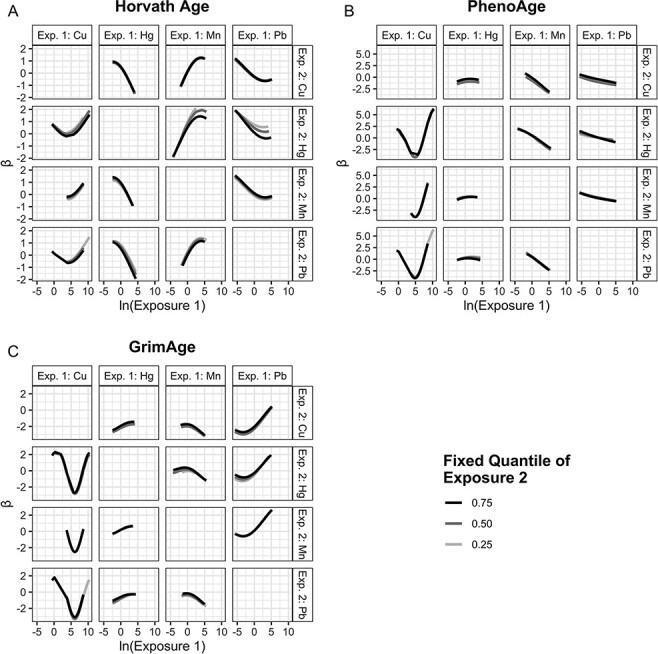
Estimated exposure–response function for accelerated (A) Horvath Age, (B) PhenoAge, and (C) GrimAge for increasing levels of each metal (Exposure 1) while holding one other metal (Exposure 2) at either the 25th, 50th, or 75th percentile and all remaining metals at the 50th percentile. As an example, the bottom left panel in 4A demonstrates the exposure–response function for increasing levels of Cu (Exposure 1) while holding Pb (Exposure 2) constant at the 25th, 50th, or 75th percentile and Mn and Hg constant at the 50th percentile. All models estimated using BKMR, include participant random effects, and control for participant sex, race, ethnicity, cigarette use, income, education, block group poverty, and Illumina platform. Exp. 1 = Exposure 1; Exp. 2 = Exposure 2

Figure 4 presents exposure–response functions for each metal (Exposure 1) and outcome while holding one other metal (Exposure 2) constant at either the 25th, 50th, or 75th percentile and all remaining metals constant at the 50th percentile. As the concentration of Hg increased from the 25th to 75th percentile, exposure–response functions between Cu, Mn, Pb, and Horvath Age acceleration (Fig. 4A) all clustered closer to the null. Similarly, the exposure–response functions of Cu and Mn on Horvath Age acceleration clustered closest to the null at the highest concentrations of Pb, while the effect of Hg on Horvath Age shifted negatively at the highest concentrations of Pb. There was very little evidence for pairwise metal interactions for either PhenoAge acceleration (Fig. 4B) or GrimAge acceleration (Fig. 4C).

In order to investigate potential interactive effects between each metal and the entire mixture, we visualized the effect of changing a single metal from the 25th to the 75th percentile while holding all other metals constant at either the 25th, 50th, or 75th percentile (Supplementary Fig. S7, *β* and 95% BCI in Supplementary Table S15). There was again relatively limited evidence of interactivity. The estimated negative effect of Hg on Horvath Age acceleration (Supplementary Fig. S7A) became increasingly negative as the rest of the mixture concentration increased, indicating that a shift of Hg from the 25th to the 75th percentile was associated with decreased Horvath Age acceleration and that this association became stronger as the concentration of all other metals increased. The effect of individual metals on PhenoAge acceleration (Supplementary Fig. S7B) showed less interactivity, with positive estimates of effect for Hg and Cu and negative estimates of effect for Mn and Pb regardless of the concentration of the other mixture components. Pb, Hg, and Cu were positively associated with GrimAge acceleration (Supplementary Fig. S7C). As the concentration of other metals decreased, the positive effects of Pb and Hg on GrimAge moved away from the null.

## Discussion

We used a community-based sample of primarily non-Hispanic African American adults living in Detroit, Michigan, from 2008 to 2013 to investigate the effects of serum ln(Pb), ln(Hg), ln(Mn), and ln(Cu) on three different scores of epigenetic age acceleration. Summarizing results from both our mixed effects and BKMR models, we identified a ∼0.25-year increase in GrimAge acceleration when increasing serum Pb from the 25th to 75th percentile and a roughly 0.2-year increase in PhenoAge acceleration when increasing serum Hg from the 25th to 75th percentile. Horvath Age acceleration was negatively associated with both toxic metals, with a roughly 0.2- and 0.4-year decrease in Horvath Age acceleration when increasing Pb and Hg from the 25th to 75th percentile, respectively. Associations between Mn, Cu, and each DNAm outcome were generally less precise than associations between Pb and Hg in our mixed effects and BKMR models, although we identified a strong curvilinear relationship between Cu and accelerated PhenoAge and GrimAge in BKMR Horvath Age acceleration decreased and PhenoAge and GrimAge acceleration increased with increasing concentrations of the entire metal mixture in our BKMR models. Although the overall statistical evidence for many of these associations was limited (most estimates of effect were relatively imprecise, and confidence intervals crossed the null), the consistency and novelty of these findings warrant interest.

Many different epigenetic clocks have been developed to estimate the DNAm age of an organism, each tapping into distinct aspects of biological aging. Horvath Age was developed using more than 30 different tissues and cell types sampled from individuals throughout the life-course and therefore constitutes an extremely accurate multi-tissue epigenetic clock [11, 16]. Despite its many strengths, Horvath Age was not specifically constructed to predict clinical phenotypes of aging (like PhenoAge [17]) or mortality (like GrimAge [18]). As such, PhenoAge and GrimAge outperform Horvath Age for the prediction of declines in health and eventual time-to-death [11, 18, 19, 48]. GrimAge is the strongest epigenetic predictor of time-to-death, as it is the only epigenetic clock explicitly designed to predict mortality in adults [18, 19, 48]. The differences between these three clocks may explain differences in the estimated joint effect of exposure to the mixture of Pb, Hg, Mn, and Cu in our BKMR models, with a net negative effect of the metal mixture on Horvath Age acceleration and a net positive effect on PhenoAge and GrimAge acceleration. Given that PhenoAge and GrimAge are strongly associated with age-related clinical phenotypes and mortality, we would expect increasing levels of the metal mixture to cause increasing PhenoAge and GrimAge acceleration due to the toxic effects of Pb and Hg within the mixture. The net negative association with Horvath Age acceleration may be due to differences in the aging-related pathways captured by this multi-tissue measure (as opposed to the blood-specific PhenoAge and GrimAge, both of which utilized clinical parameters beyond age alone in their construction) or due to its comparatively weaker associations with mortality and the clinical phenotypes captured by PhenoAge and GrimAge [17, 18]. Similarly, the strikingly curvilinear relationship between serum Cu and accelerated PhenoAge and GrimAge may highlight the potentially negative health risks of Cu (a biologically essential metal) concentrations that are either too low or too high for normal physiologic function. This narrative most clearly fits with our BKMR findings for GrimAge acceleration, which demonstrated univariate exposure–response functions for Pb (increasing GrimAge acceleration with increasing Pb), Hg (increasing GrimAge acceleration with increasing Hg), and Cu (increasing GrimAge acceleration with low or high Cu) that align neatly with expectations (Mn appeared unrelated to GrimAge acceleration).

Research in the Veterans Affairs Normative Aging Study found that urinary Mn (but not urinary arsenic, cadmium, Pb, or Hg) was strongly associated with increasing PhenoAge acceleration using BKMR [49] but included only 48 participants with one observation per participant. Xiao *et al.* measured 22 blood metals (including Mn, Cu, and Pb) among 288 participants greater than 50 years of age in Guangxi, China, finding that increasing joint exposure to non-essential metals was associated with increased Horvath Age acceleration and increasing joint exposure to essential metals was associated with decreased Horvath Age acceleration [50]. Joint exposure metrics for PhenoAge and GrimAge were not presented, however, making it difficult to directly compare our findings with those DNAm clocks most predictive of mortality [50]. Other studies of metals and DNAm age are limited by even smaller cohorts and the number of metal exposures [51, 52].

Our approach highlights the strength of BKMR as a tool to evaluate non-linear and interactive effects of exposure mixtures, an area of research that Fry and Martin mentioned as critical to advance the field of environmental epigenetics [14]. Notably, we find general concordance between our single-metal mixed effects models and our BKMR results (e.g. positive associations between Pb and GrimAge acceleration, positive associations between Hg and PhenoAge acceleration, negative associations between Hg and Horvath Age acceleration, etc.), but BKMR allows for the estimation and visualization of non-linear and interactive effects without over-parameterizing a traditional regression model [42, 43]. This allows us to uncover, for example, the curvilinear relationship between Cu and PhenoAge and GrimAge acceleration without explicitly modeling such an effect using splines or another standard regression tool. Ultimately, including multiple regression techniques allows us to interrogate single-metal effects and mixture effects from a variety of angles, lending additional credence to findings that match between the two approaches.

Our approach is limited in several key ways. First and foremost, selection bias induced by loss-to-follow up and the voluntary provision of biospecimens for metal testing and DNAm assessment may lead to a non-representative cohort of participants in our analytic models [53]. Although the DNHS was representative of the population of Detroit at the time of initial sampling in Wave 1 (2008) [28, 29], participants included in our analysis are different from the Detroit population along axes of race, ethnicity, household income, lifetime smoking, and any number of other unobserved characteristics. While much of this difference is likely due to the intentional over-selection of non-Hispanic Black/African Americans for DNAm assessment (a considerable strength, given how few genomic studies have historically included participants of diverse racial and ethnic self-identification [54]), we are unable to disentangle intentional selection from potential analytic bias due to selection in our cohort. The small number of DNHS participants with measured serum metals and DNAm also decreases our analytic sample size and power, leading to relatively wide confidence intervals and limiting our ability to draw robust statistical conclusions from the available data. The differences between our analytic cohort and the Detroit-representative DNHS cohort are small based on observable characteristics (limited to slight differences in income, age, and lifetime smoking); however, making it unlikely that major selection bias plays a part in our findings.

Although the inclusion of multiple metals (both essential and non-essential) is a strength of our approach, we by no means include all metals (or other potentially correlated environmental toxicants) that may impact the epigenome or epigenetic aging. Future studies should certainly include as many metal exposures as possible, given that BKMR and other environmental mixtures approaches, such as quantile G-computation [55], can include high-dimensional mixtures data to estimate the effect of joint environmental exposures. The development of mixture modeling tools and high-throughput assays for the rapid assessment of multiple environmental toxicants in biospecimens is critical to make this approach feasible in large, longitudinal, population-based studies such as the DNHS. Our approach is also limited because we tested metals in serum rather than blood. Blood metal levels are more commonly used in the literature and allow easy comparison with blood reference levels (e.g. Centers for Disease Control and Prevention blood Pb standards [4], etc.). The distributions of serum Hg, Mn, and Cu in our cohort are consistent with published laboratory reference levels, however [45-47].

The use of different pre-processing procedures for the 450K and 850K DNAm data included in our analysis is another limitation of our approach. The use of different DNAm processing pipelines is extremely common in the literature, however (including the development of PhenoAge [17] and GrimAge [18]), and should only change the intercept (not the regression coefficient) in any given model [37]. We adjust for platform in all of our models, so any effects of DNAm array (based on design and/or processing) should be extremely minimal.

We used longitudinal data from a population of adults living in Detroit, Michigan, to evaluate the effects of four serum metals on three different markers of DNAm aging. To our knowledge, this is the first study to demonstrate a net positive relationship between the total metal mixture (Pb, Hg, Mn, and Cu) and accelerated PhenoAge and GrimAge, two robust markers of all-cause mortality [17, 18]. Cu demonstrated a strong U-shaped relationship with both PhenoAge and GrimAge acceleration. Pb was positively associated with GrimAge acceleration, and Hg was positively associated with PhenoAge acceleration in both mixed effects and BKMR models, with an increase of each metal from the 25th to 75th percentile adding ∼0.25 years to the respective epigenetic clock. These results suggest that low-level, population-wide exposure to toxic metals such as Pb and Hg may contribute to accelerated biological aging in the US population. Future research should replicate and expand this work with a larger number of metals (both essential and non-essential) to evaluate the effect of simultaneous metal exposures on DNAm aging.

## Supplementary Material

dvac018_Supp

## Data Availability

The DNHS data used for this analysis are not publicly available due to the inclusion of potentially identifiable information. Please contact Dr Allison E. Aiello (project Principal Investigator) for more information regarding access to DNHS data.
