# Supplementary material for: Serum lead, mercury, manganese, and copper and DNA methylation age among adults in Detroit, Michigan
Source: Environ Epigenet. 2022 Sep 21;8(1):dvac018. doi: 10.1093/eep/dvac018 (PMC9620967; doi:10.1093/eep/dvac018)
Supplement: dvac018_Supp [file dvac018_supp.zip › suppl_data/Lodge_Aim_3_Supplemental_Images_and_Tables_rev2.docx]

**Supplemental Table 1.** Percentage of participants providing venous blood samples in each DNHS wave. Note that DNHS wave 5 was *a priori* limited to participants who had previously provided at least one biospecimen.

| **DNHS Wave** | **Total Participants** | **Biospecimens Collected** | **% Providing Biospecimens** |
| --- | --- | --- | --- |
| Wave 1 | 1547 | 612 | 39.6 % |
| Wave 2 | 1588 | 803 | 50.6 % |
| Wave 4 | 845 | 503 | 59.5 % |
| Wave 5 | 353 | 230 | 65.2 % |

**Supplemental Table 2.** ICP-MS parameters for the measurement of Pb, Hg, Mn, and Cu in serum. All parameters were optimized as defined by the manufacturer prior to analysis.

| **Component/Parameter** | **Value** |
| --- | --- |
| Plasma Gas Flow | 18.0 L/min |
| Auxiliary Gas Flow | 1.20 L/min |
| Nebulizer Gas Flow | 0.96 L/min |
| Sample Uptake Rate | 200 μL/min |
| RF Power | 1650 W |
| Cone | Nickel |
| Analytes | ^208^Pb, ^202^Hg, ^55^Mn, ^63^Cu |
| Internal Standards | ^89^Y, ^209^Bi |
| Replicates | 5 |

**Supplemental Table 3.** ICP-MS Limits of Detection (LOD) and percent of samples below the LOD for serum Pb, Hg, Mn, and Cu.

| **Serum metal** | **LOD** | **% below LOD** |
| --- | --- | --- |
| ^208^Pb | 0.005 ug/L | 1.2 % |
| ^202^Hg | 0.124 ug/L | 12.3 % |
| ^55^Mn | 0.020 ug/L | 0.7 % |
| ^63^Cu | 0.498 ug/L | 0.2 % |

**Supplemental Table 4.** 10^th^, 15^th^, 20^th^, 25^th^, 50^th^, 75^th^, 80^th^, 85^th^, and 90^th^ percentiles of serum Pb, Hg, Mn, Cu, ln(Pb), ln(Hg), ln(Mn), and ln(Cu) (all variables in ug/L).

| **Percentile** | **Pb** | **Hg** | **Mn** | **Cu** | **ln(Pb)** | **ln(Hg)** | **ln(Mn)** | **ln(Cu)** |
| --- | --- | --- | --- | --- | --- | --- | --- | --- |
| 10% | 0.094 | 0.088 | 0.881 | 937.285 | -2.360 | -2.433 | -0.127 | 6.843 |
| 15% | 0.120 | 0.203 | 0.952 | 989.120 | -2.121 | -1.597 | -0.049 | 6.897 |
| 20% | 0.142 | 0.272 | 1.001 | 1026.428 | -1.951 | -1.302 | 0.001 | 6.934 |
| 25% | 0.163 | 0.331 | 1.057 | 1067.333 | -1.814 | -1.106 | 0.055 | 6.973 |
| 50% | 0.246 | 0.744 | 1.330 | 1250.375 | -1.401 | -0.296 | 0.285 | 7.131 |
| 75% | 0.400 | 1.696 | 1.776 | 1451.763 | -0.916 | 0.528 | 0.574 | 7.281 |
| 80% | 0.455 | 2.033 | 1.888 | 1529.469 | -0.787 | 0.710 | 0.636 | 7.333 |
| 85% | 0.564 | 2.706 | 2.031 | 1589.395 | -0.573 | 0.995 | 0.708 | 7.371 |
| 90% | 0.673 | 3.850 | 2.268 | 1690.574 | -0.397 | 1.348 | 0.819 | 7.433 |

**Supplemental Table 5.** Number of participants at each categorical exposure level of serum ln(Pb), ln(Hg), ln(Mn), and ln(Cu). The “Exposed” column refers to participants in the upper tertile or upper/lower 10^th^, 15^th^, or 20^th^ percentile of each metal (depending on the coding in the “Variable” column). The “Unexposed” column refers to all other participants.

| **Variable** | **Exposed** | **Unexposed** | **Missing** |
| --- | --- | --- | --- |
| Upper Tertile Pb | 166 | 331 | 0 |
| Upper Tertile Hg | 162 | 324 | 11 |
| Upper/Lower 10^th^ Percentile Mn | 100 | 397 | 0 |
| Upper/Lower 10^th^ Percentile Cu | 100 | 397 | 0 |
| Upper/Lower 15^th^ Percentile Mn | 155 | 342 | 0 |
| Upper/Lower 15^th^ Percentile Cu | 150 | 347 | 0 |
| Upper/Lower 20^th^ Percentile Mn | 200 | 297 | 0 |
| Upper/Lower 20^th^ Percentile Cu | 200 | 297 | 0 |

**Supplemental Figure 1.** Correlations between participant chronological age and (A) Horvath Age, (B) PhenoAge, and (C) GrimAge in all 497 samples in our analytic cohort, with trend lines displayed in red. All three measurements of DNAm age are extremely well correlated with chronological age.


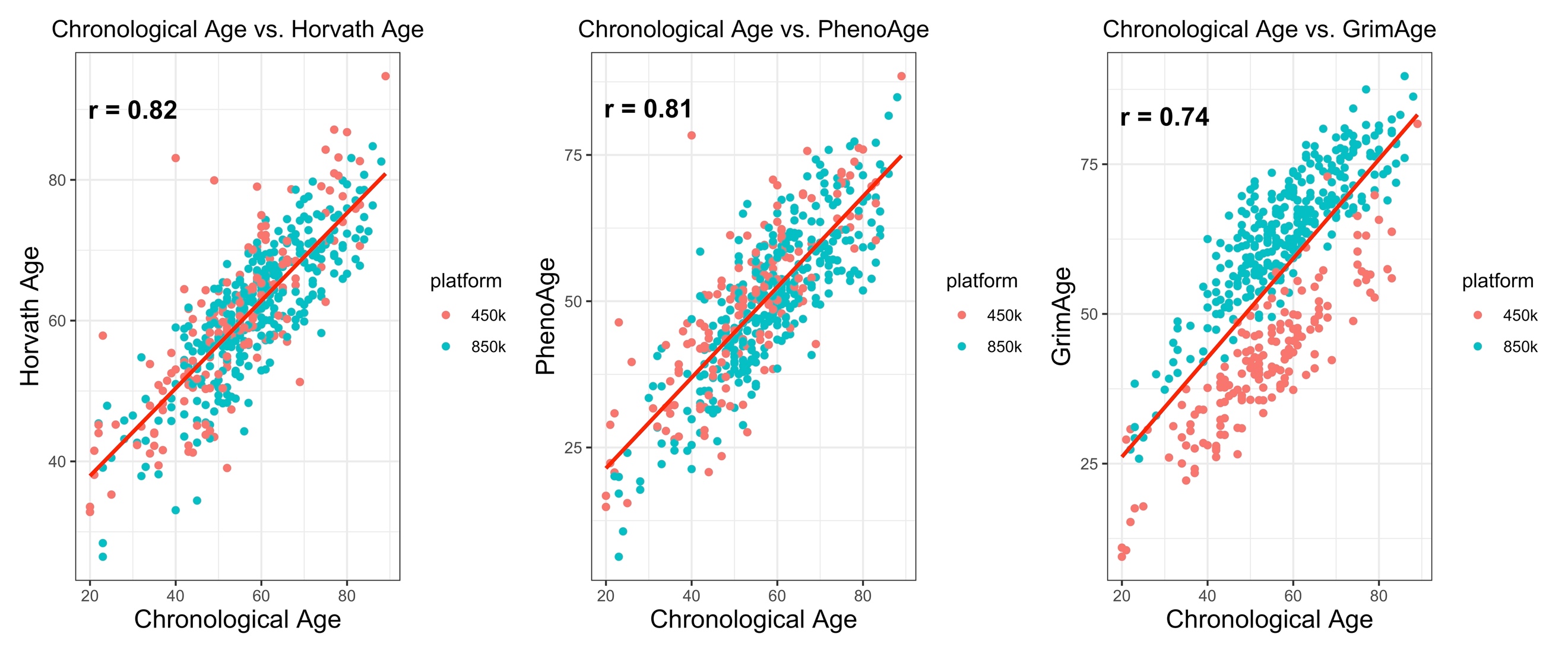


**Supplemental Figure 2.** Correlations between (A) Horvath Age and PhenoAge, (B) Horvath Age and GrimAge, and (C) PhenoAge and GrimAge in all 497 samples in our analytic cohort, with trend lines displayed in red. All three measurements of DNAm age are extremely well correlated with each other.


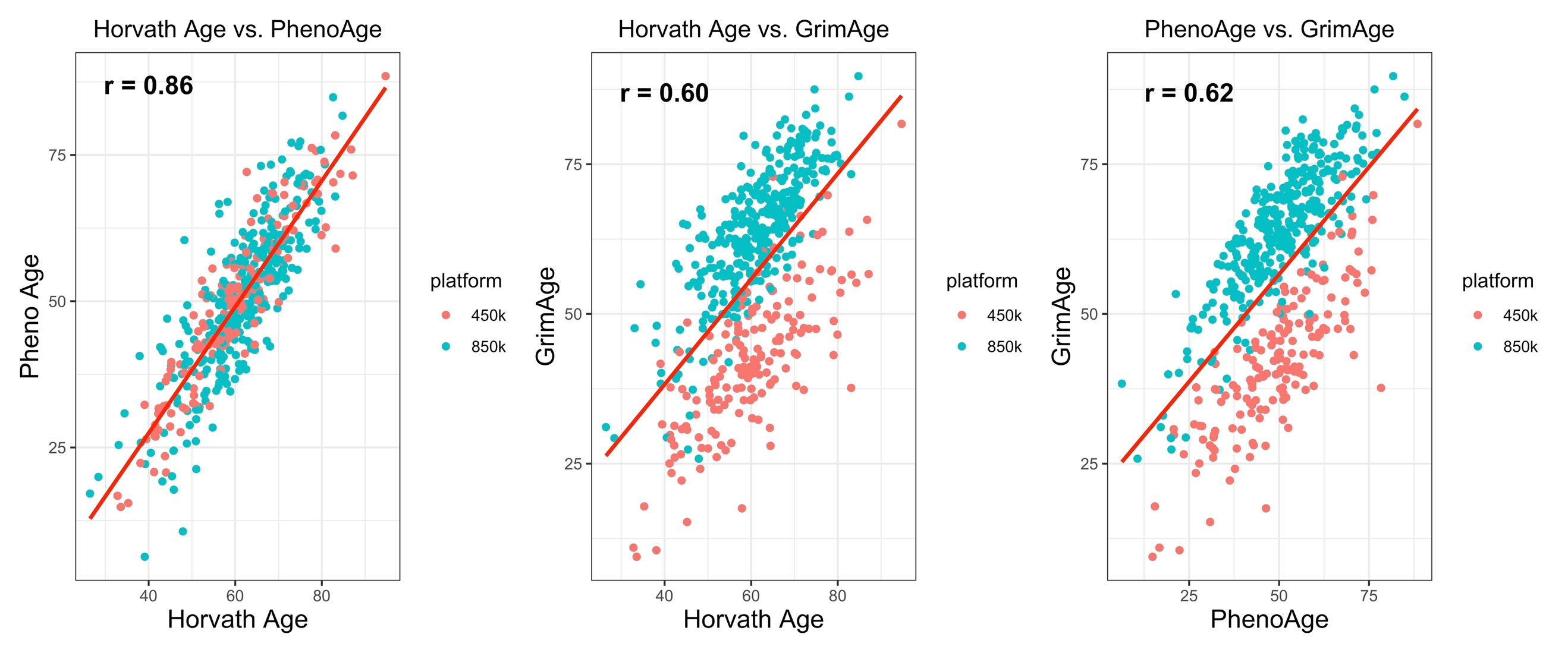


**Supplemental Figure 3.** Exclusion criteria for this analysis. 2081 DNHS participants ever completed at least one survey, with 5672 surveys completed over five years. 497 surveys from 290 unique participants are included in this analysis.


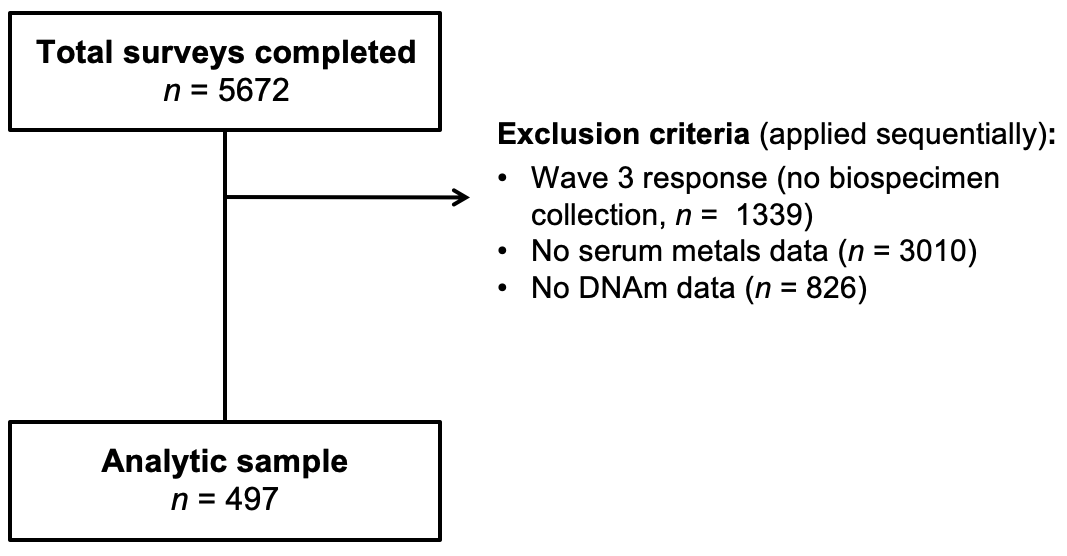


**Supplemental Figure 4.** Directed Acyclic Graph (DAG) depicting causal relationships between variables included in our models.


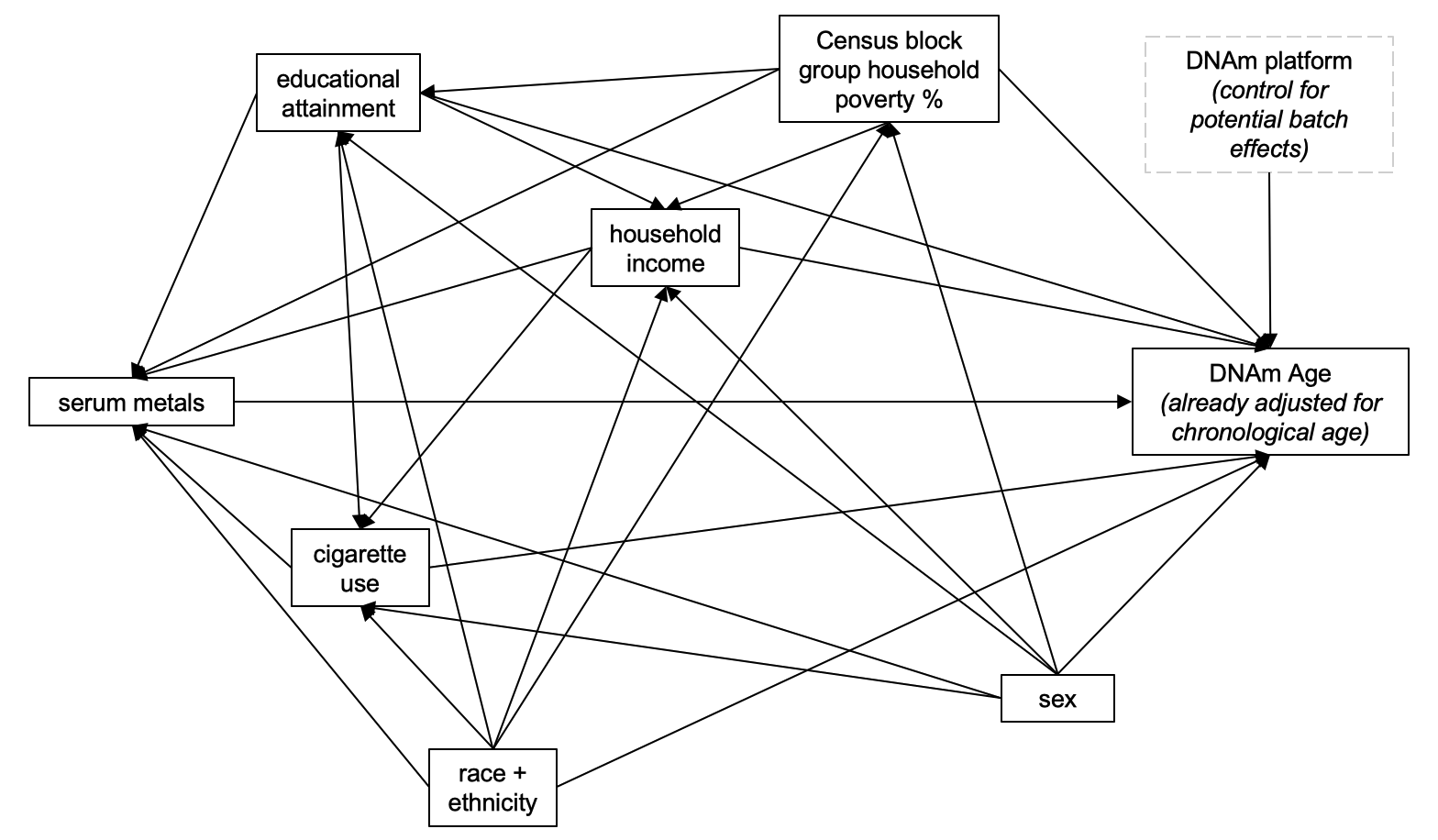


**Supplemental Table 6.** Associations between linear natural-log transformed serum Pb and Hg and upper tertile (versus middle or lower tertile) serum Pb and Hg and accelerated Horvath Age, PhenoAge, and GrimAge. All models include participant random effects and control for participant sex, race, ethnicity, cigarette use, income, education, block group poverty and Illumina platform.

|  | | | | | | | | | | | | |
| --- | --- | --- | --- | --- | --- | --- | --- | --- | --- | --- | --- | --- |
|  | Dependent variable: | | | | | | | | | | | |
|  |  | | | | | | | | | | | |
|  | Horvath Age | Pheno Age | Grim Age | Horvath Age | Pheno Age | Grim Age | Horvath Age | Pheno Age | Grim Age | Horvath Age | Pheno Age | Grim Age |
|  | (1) | (2) | (3) | (4) | (5) | (6) | (7) | (8) | (9) | (10) | (11) | (12) |
|  | | | | | | | | | | | | |
| Linear Serum ln(Pb) | -0.08 | -0.09 | 0.16 |  |  |  |  |  |  |  |  |  |
|  | (-0.51, 0.34) | (-0.57, 0.40) | (-0.11, 0.43) |  |  |  |  |  |  |  |  |  |
|  |  |  |  |  |  |  |  |  |  |  |  |  |
| Elevated Serum Pb (highest tertile) |  |  |  | -0.38 | -0.12 | 0.46 |  |  |  |  |  |  |
|  |  |  |  | (-1.34, 0.57) | (-1.21, 0.98) | (-0.14, 1.07) |  |  |  |  |  |  |
|  |  |  |  |  |  |  |  |  |  |  |  |  |
| Linear Serum ln(Hg) |  |  |  |  |  |  | -0.17 | 0.10 | -0.04 |  |  |  |
|  |  |  |  |  |  |  | (-0.54, 0.20) | (-0.33, 0.53) | (-0.28, 0.21) |  |  |  |
|  |  |  |  |  |  |  |  |  |  |  |  |  |
| Elevated Serum Hg (highest tertile) |  |  |  |  |  |  |  |  |  | -0.71 | 0.10 | 0.01 |
|  |  |  |  |  |  |  |  |  |  | (-1.69, 0.26) | (-1.04, 1.24) | (-0.62, 0.64) |
|  |  |  |  |  |  |  |  |  |  |  |  |  |
| Female | -0.25 | 0.02 | -1.94^**^ | -0.27 | 0.02 | -1.92^**^ | -0.14 | 0.05 | -1.95^**^ | -0.13 | 0.05 | -1.95^**^ |
|  | (-1.65, 1.14) | (-1.80, 1.83) | (-3.12, -0.75) | (-1.67, 1.12) | (-1.80, 1.83) | (-3.11, -0.74) | (-1.55, 1.27) | (-1.80, 1.89) | (-3.14, -0.75) | (-1.53, 1.28) | (-1.79, 1.89) | (-3.15, -0.75) |
|  |  |  |  |  |  |  |  |  |  |  |  |  |
| Race/Eth: non-Hisp. Afr. Am. | -0.20 | 1.50 | 1.37 | -0.21 | 1.50 | 1.38 | -0.23 | 1.50 | 1.29 | -0.21 | 1.48 | 1.30 |
|  | (-2.55, 2.16) | (-1.53, 4.54) | (-0.60, 3.33) | (-2.57, 2.14) | (-1.53, 4.54) | (-0.58, 3.35) | (-2.63, 2.17) | (-1.62, 4.62) | (-0.72, 3.30) | (-2.60, 2.19) | (-1.64, 4.59) | (-0.70, 3.31) |
|  |  |  |  |  |  |  |  |  |  |  |  |  |
| Race/Eth: Other | -0.73 | 1.01 | -0.12 | -0.77 | 1.01 | -0.08 | -0.75 | 0.97 | -0.25 | -0.76 | 0.96 | -0.24 |
|  | (-5.78, 4.32) | (-5.55, 7.58) | (-4.42, 4.17) | (-5.82, 4.28) | (-5.56, 7.58) | (-4.36, 4.21) | (-5.83, 4.33) | (-5.68, 7.63) | (-4.57, 4.08) | (-5.84, 4.32) | (-5.69, 7.61) | (-4.56, 4.08) |
|  |  |  |  |  |  |  |  |  |  |  |  |  |
| Ever Smoker | 0.36 | 1.84 | 4.37^***^ | 0.38 | 1.84 | 4.35^***^ | 0.37 | 1.68 | 4.35^***^ | 0.38 | 1.71 | 4.33^***^ |
|  | (-1.19, 1.92) | (-0.17, 3.86) | (3.05, 5.68) | (-1.18, 1.94) | (-0.18, 3.87) | (3.03, 5.67) | (-1.21, 1.95) | (-0.38, 3.75) | (3.01, 5.69) | (-1.20, 1.96) | (-0.35, 3.77) | (3.00, 5.67) |
|  |  |  |  |  |  |  |  |  |  |  |  |  |
| Income: >= 25k | -0.22 | 0.34 | -1.02^*^ | -0.22 | 0.34 | -1.02^*^ | -0.45 | 0.32 | -1.02^*^ | -0.43 | 0.32 | -1.02^*^ |
|  | (-1.47, 1.04) | (-1.19, 1.86) | (-1.92, -0.12) | (-1.47, 1.04) | (-1.19, 1.87) | (-1.92, -0.11) | (-1.71, 0.82) | (-1.23, 1.88) | (-1.94, -0.10) | (-1.69, 0.83) | (-1.23, 1.88) | (-1.94, -0.10) |
|  |  |  |  |  |  |  |  |  |  |  |  |  |
| Ed: > H.S. Grad/Equiv | 0.48 | 0.01 | -0.65 | 0.48 | -0.00 | -0.64 | 0.43 | 0.18 | -0.63 | 0.39 | 0.16 | -0.62 |
|  | (-0.85, 1.82) | (-1.69, 1.70) | (-1.71, 0.41) | (-0.85, 1.82) | (-1.69, 1.69) | (-1.70, 0.42) | (-0.94, 1.79) | (-1.57, 1.93) | (-1.73, 0.47) | (-0.97, 1.76) | (-1.59, 1.91) | (-1.72, 0.49) |
|  |  |  |  |  |  |  |  |  |  |  |  |  |
| Blk. Grp. Poverty % | 0.00 | 0.01 | 0.01 | 0.00 | 0.01 | 0.01 | 0.00 | 0.01 | 0.01 | 0.00 | 0.01 | 0.01 |
|  | (-0.03, 0.04) | (-0.03, 0.06) | (-0.01, 0.04) | (-0.03, 0.04) | (-0.03, 0.06) | (-0.01, 0.04) | (-0.03, 0.04) | (-0.03, 0.06) | (-0.02, 0.04) | (-0.03, 0.04) | (-0.03, 0.06) | (-0.02, 0.04) |
|  |  |  |  |  |  |  |  |  |  |  |  |  |
| Platform: 850K | -0.52 | -0.28 | 0.01 | -0.53 | -0.29 | 0.04 | -0.60 | -0.39 | -0.04 | -0.60 | -0.41 | -0.03 |
|  | (-1.58, 0.53) | (-1.53, 0.96) | (-0.70, 0.72) | (-1.58, 0.53) | (-1.53, 0.96) | (-0.67, 0.75) | (-1.68, 0.47) | (-1.67, 0.89) | (-0.77, 0.69) | (-1.67, 0.47) | (-1.69, 0.87) | (-0.76, 0.70) |
|  |  |  |  |  |  |  |  |  |  |  |  |  |
| Constant | -0.14 | -3.30 | -2.80^*^ | 0.10 | -3.16 | -3.16^*^ | 0.02 | -3.09 | -2.80^*^ | 0.23 | -3.15 | -2.79^*^ |
|  | (-3.35, 3.08) | (-7.39, 0.80) | (-5.41, -0.19) | (-3.10, 3.30) | (-7.25, 0.93) | (-5.77, -0.56) | (-3.22, 3.25) | (-7.25, 1.06) | (-5.44, -0.15) | (-3.00, 3.46) | (-7.31, 1.01) | (-5.43, -0.14) |
|  |  |  |  |  |  |  |  |  |  |  |  |  |
|  | | | | | | | | | | | | |
| Observations | 478 | 478 | 478 | 478 | 478 | 478 | 467 | 467 | 467 | 467 | 467 | 467 |
|  | | | | | | | | | | | | |
| Note: | ^*^p<0.05, ^**^p<0.01, ^***^p<0.001 | | | | | | | | | | | |

**Supplemental Table 7.** Associations between linear natural-log transformed serum Mn and Cu and upper/lower 15^th^ percentile (versus 16^th^-84^th^ percentile) serum Mn and Cu and accelerated Horvath Age, PhenoAge, and GrimAge. All models include participant random effects and control for participant sex, race, ethnicity, cigarette use, income, education, block group poverty and Illumina platform.

|  | | | | | | | | | | | | |
| --- | --- | --- | --- | --- | --- | --- | --- | --- | --- | --- | --- | --- |
|  | Dependent variable: | | | | | | | | | | | |
|  |  | | | | | | | | | | | |
|  | Horvath Age | Pheno Age | Grim Age | Horvath Age | Pheno Age | Grim Age | Horvath Age | Pheno Age | Grim Age | Horvath Age | Pheno Age | Grim Age |
|  | (1) | (2) | (3) | (4) | (5) | (6) | (7) | (8) | (9) | (10) | (11) | (12) |
|  | | | | | | | | | | | | |
| Linear Serum ln(Mn) | -0.38 | -0.20 | 0.25 |  |  |  |  |  |  |  |  |  |
|  | (-1.10, 0.35) | (-1.05, 0.66) | (-0.24, 0.73) |  |  |  |  |  |  |  |  |  |
|  |  |  |  |  |  |  |  |  |  |  |  |  |
| Abnormal Serum Mn (highest/lowest 15%tile) |  |  |  | -0.13 | -0.82 | 0.27 |  |  |  |  |  |  |
|  |  |  |  | (-1.13, 0.86) | (-1.96, 0.33) | (-0.37, 0.91) |  |  |  |  |  |  |
|  |  |  |  |  |  |  |  |  |  |  |  |  |
| Linear Serum ln(Cu) |  |  |  |  |  |  | -0.23 | -0.02 | -0.18 |  |  |  |
|  |  |  |  |  |  |  | (-1.06, 0.60) | (-0.98, 0.93) | (-0.71, 0.35) |  |  |  |
|  |  |  |  |  |  |  |  |  |  |  |  |  |
| Abnormal Serum Cu (highest/lowest 15%tile) |  |  |  |  |  |  |  |  |  | -0.37 | 0.38 | -0.10 |
|  |  |  |  |  |  |  |  |  |  | (-1.39, 0.65) | (-0.80, 1.55) | (-0.75, 0.56) |
|  |  |  |  |  |  |  |  |  |  |  |  |  |
| Female | -0.21 | 0.04 | -1.97^**^ | -0.24 | 0.04 | -1.96^**^ | -0.21 | 0.03 | -1.93^**^ | -0.26 | 0.04 | -1.96^**^ |
|  | (-1.62, 1.19) | (-1.77, 1.86) | (-3.16, -0.79) | (-1.64, 1.15) | (-1.78, 1.86) | (-3.14, -0.78) | (-1.62, 1.19) | (-1.79, 1.85) | (-3.12, -0.74) | (-1.66, 1.14) | (-1.77, 1.86) | (-3.14, -0.78) |
|  |  |  |  |  |  |  |  |  |  |  |  |  |
| Race/Eth: non-Hisp. Afr. Am. | -0.23 | 1.48 | 1.40 | -0.20 | 1.42 | 1.39 | -0.14 | 1.51 | 1.40 | -0.22 | 1.53 | 1.35 |
|  | (-2.60, 2.14) | (-1.56, 4.52) | (-0.57, 3.36) | (-2.57, 2.16) | (-1.62, 4.47) | (-0.57, 3.36) | (-2.51, 2.23) | (-1.53, 4.56) | (-0.57, 3.36) | (-2.58, 2.14) | (-1.50, 4.57) | (-0.61, 3.32) |
|  |  |  |  |  |  |  |  |  |  |  |  |  |
| Race/Eth: Other | -0.79 | 0.98 | -0.09 | -0.75 | 0.80 | -0.07 | -0.69 | 1.03 | -0.14 | -0.77 | 1.08 | -0.16 |
|  | (-5.87, 4.29) | (-5.59, 7.56) | (-4.38, 4.21) | (-5.82, 4.31) | (-5.79, 7.40) | (-4.36, 4.22) | (-5.76, 4.37) | (-5.54, 7.60) | (-4.43, 4.15) | (-5.83, 4.29) | (-5.50, 7.66) | (-4.45, 4.13) |
|  |  |  |  |  |  |  |  |  |  |  |  |  |
| Ever Smoker | 0.35 | 1.83 | 4.39^***^ | 0.36 | 1.87 | 4.37^***^ | 0.36 | 1.84 | 4.39^***^ | 0.37 | 1.82 | 4.39^***^ |
|  | (-1.21, 1.91) | (-0.19, 3.85) | (3.07, 5.70) | (-1.20, 1.92) | (-0.16, 3.89) | (3.06, 5.69) | (-1.20, 1.91) | (-0.18, 3.86) | (3.07, 5.70) | (-1.19, 1.92) | (-0.20, 3.84) | (3.07, 5.71) |
|  |  |  |  |  |  |  |  |  |  |  |  |  |
| Income: >= 25k | -0.18 | 0.35 | -1.04^*^ | -0.22 | 0.30 | -1.02^*^ | -0.20 | 0.34 | -1.01^*^ | -0.18 | 0.30 | -1.02^*^ |
|  | (-1.44, 1.07) | (-1.17, 1.88) | (-1.95, -0.14) | (-1.48, 1.04) | (-1.23, 1.82) | (-1.92, -0.11) | (-1.45, 1.06) | (-1.19, 1.87) | (-1.92, -0.11) | (-1.44, 1.08) | (-1.23, 1.83) | (-1.93, -0.11) |
|  |  |  |  |  |  |  |  |  |  |  |  |  |
| Ed: > H.S. Grad/Equiv | 0.51 | 0.02 | -0.65 | 0.48 | 0.04 | -0.63 | 0.47 | -0.01 | -0.62 | 0.47 | -0.01 | -0.61 |
|  | (-0.83, 1.86) | (-1.68, 1.71) | (-1.71, 0.41) | (-0.86, 1.82) | (-1.65, 1.74) | (-1.69, 0.43) | (-0.87, 1.81) | (-1.70, 1.69) | (-1.68, 0.44) | (-0.86, 1.81) | (-1.70, 1.69) | (-1.67, 0.45) |
|  |  |  |  |  |  |  |  |  |  |  |  |  |
| Blk. Grp. Poverty % | 0.00 | 0.01 | 0.01 | 0.00 | 0.01 | 0.01 | 0.00 | 0.01 | 0.01 | 0.00 | 0.01 | 0.01 |
|  | (-0.03, 0.04) | (-0.03, 0.06) | (-0.01, 0.04) | (-0.03, 0.04) | (-0.03, 0.06) | (-0.02, 0.04) | (-0.03, 0.04) | (-0.03, 0.06) | (-0.02, 0.04) | (-0.03, 0.04) | (-0.03, 0.06) | (-0.02, 0.04) |
|  |  |  |  |  |  |  |  |  |  |  |  |  |
| Platform: 850K | -0.58 | -0.31 | 0.05 | -0.51 | -0.21 | -0.02 | -0.54 | -0.29 | 0.01 | -0.50 | -0.31 | 0.02 |
|  | (-1.64, 0.47) | (-1.56, 0.94) | (-0.66, 0.76) | (-1.57, 0.54) | (-1.45, 1.03) | (-0.73, 0.70) | (-1.60, 0.51) | (-1.53, 0.96) | (-0.70, 0.72) | (-1.55, 0.56) | (-1.56, 0.93) | (-0.69, 0.73) |
|  |  |  |  |  |  |  |  |  |  |  |  |  |
| Constant | 0.14 | -3.10 | -3.11^*^ | -0.01 | -2.94 | -3.06^*^ | 1.55 | -3.06 | -1.73 | 0.03 | -3.29 | -2.95^*^ |
|  | (-3.07, 3.36) | (-7.19, 0.99) | (-5.72, -0.50) | (-3.21, 3.19) | (-7.02, 1.15) | (-5.66, -0.46) | (-4.98, 8.08) | (-10.78, 4.66) | (-6.21, 2.75) | (-3.16, 3.22) | (-7.36, 0.79) | (-5.55, -0.35) |
|  |  |  |  |  |  |  |  |  |  |  |  |  |
|  | | | | | | | | | | | | |
| Observations | 478 | 478 | 478 | 478 | 478 | 478 | 478 | 478 | 478 | 478 | 478 | 478 |
|  | | | | | | | | | | | | |
| Note: | ^*^p<0.05, ^**^p<0.01, ^***^p<0.001 | | | | | | | | | | | |

**Supplemental Table 8.** Associations between upper/lower 10^th^ percentile (versus 11^th^-89^th^ percentile) or 20^th^ percentile (versus 21^st^-79^th^ percentile) serum Mn and Cu and accelerated Horvath Age, PhenoAge, and GrimAge. All models include participant random effects and control for participant sex, race, ethnicity, cigarette use, income, education, block group poverty and Illumina platform.

|  | | | | | | | | | | | | |
| --- | --- | --- | --- | --- | --- | --- | --- | --- | --- | --- | --- | --- |
|  | Dependent variable: | | | | | | | | | | | |
|  |  | | | | | | | | | | | |
|  | Horvath Age | Pheno Age | Grim Age | Horvath Age | Pheno Age | Grim Age | Horvath Age | Pheno Age | Grim Age | Horvath Age | Pheno Age | Grim Age |
|  | (1) | (2) | (3) | (4) | (5) | (6) | (7) | (8) | (9) | (10) | (11) | (12) |
|  | | | | | | | | | | | | |
| Abnormal Serum Mn (highest/lowest 10%tile) | 0.30 | -0.57 | 0.34 |  |  |  |  |  |  |  |  |  |
|  | (-0.83, 1.43) | (-1.86, 0.72) | (-0.38, 1.06) |  |  |  |  |  |  |  |  |  |
|  |  |  |  |  |  |  |  |  |  |  |  |  |
| Abnormal Serum Mn (highest/lowest 20%tile) |  |  |  | -0.30 | -0.59 | 0.28 |  |  |  |  |  |  |
|  |  |  |  | (-1.23, 0.63) | (-1.66, 0.48) | (-0.32, 0.88) |  |  |  |  |  |  |
|  |  |  |  |  |  |  |  |  |  |  |  |  |
| Abnormal Serum Cu (highest/lowest 10%tile) |  |  |  |  |  |  | -1.04 | -0.55 | 0.04 |  |  |  |
|  |  |  |  |  |  |  | (-2.22, 0.14) | (-1.92, 0.81) | (-0.73, 0.80) |  |  |  |
|  |  |  |  |  |  |  |  |  |  |  |  |  |
| Abnormal Serum Cu (highest/lowest 20%tile) |  |  |  |  |  |  |  |  |  | 0.01 | 0.66 | -0.11 |
|  |  |  |  |  |  |  |  |  |  | (-0.96, 0.97) | (-0.44, 1.77) | (-0.73, 0.51) |
|  |  |  |  |  |  |  |  |  |  |  |  |  |
| Female | -0.26 | 0.05 | -1.97^**^ | -0.23 | 0.05 | -1.97^**^ | -0.31 | -0.01 | -1.95^**^ | -0.24 | 0.05 | -1.96^**^ |
|  | (-1.65, 1.14) | (-1.76, 1.87) | (-3.15, -0.79) | (-1.63, 1.16) | (-1.77, 1.87) | (-3.15, -0.78) | (-1.70, 1.09) | (-1.82, 1.81) | (-3.14, -0.77) | (-1.64, 1.15) | (-1.77, 1.87) | (-3.14, -0.78) |
|  |  |  |  |  |  |  |  |  |  |  |  |  |
| Race/Eth: non-Hisp. Afr. Am. | -0.16 | 1.44 | 1.41 | -0.20 | 1.49 | 1.38 | -0.29 | 1.46 | 1.36 | -0.19 | 1.50 | 1.36 |
|  | (-2.52, 2.21) | (-1.61, 4.48) | (-0.56, 3.37) | (-2.56, 2.16) | (-1.56, 4.53) | (-0.59, 3.34) | (-2.65, 2.07) | (-1.58, 4.50) | (-0.60, 3.33) | (-2.55, 2.17) | (-1.54, 4.54) | (-0.60, 3.33) |
|  |  |  |  |  |  |  |  |  |  |  |  |  |
| Race/Eth: Other | -0.66 | 0.92 | -0.08 | -0.79 | 0.88 | -0.07 | -0.95 | 0.90 | -0.14 | -0.72 | 0.96 | -0.13 |
|  | (-5.72, 4.39) | (-5.66, 7.50) | (-4.37, 4.21) | (-5.86, 4.28) | (-5.72, 7.48) | (-4.36, 4.22) | (-6.01, 4.11) | (-5.67, 7.48) | (-4.43, 4.15) | (-5.77, 4.34) | (-5.62, 7.54) | (-4.42, 4.15) |
|  |  |  |  |  |  |  |  |  |  |  |  |  |
| Ever Smoker | 0.34 | 1.86 | 4.37^***^ | 0.36 | 1.85 | 4.37^***^ | 0.38 | 1.85 | 4.38^***^ | 0.35 | 1.83 | 4.39^***^ |
|  | (-1.22, 1.89) | (-0.16, 3.89) | (3.05, 5.68) | (-1.20, 1.92) | (-0.17, 3.88) | (3.06, 5.69) | (-1.17, 1.94) | (-0.16, 3.87) | (3.07, 5.70) | (-1.20, 1.91) | (-0.19, 3.85) | (3.07, 5.70) |
|  |  |  |  |  |  |  |  |  |  |  |  |  |
| Income: >= 25k | -0.20 | 0.32 | -1.02^*^ | -0.22 | 0.33 | -1.03^*^ | -0.17 | 0.37 | -1.03^*^ | -0.21 | 0.28 | -1.02^*^ |
|  | (-1.46, 1.06) | (-1.21, 1.85) | (-1.93, -0.12) | (-1.47, 1.04) | (-1.20, 1.85) | (-1.93, -0.12) | (-1.42, 1.09) | (-1.16, 1.90) | (-1.94, -0.13) | (-1.47, 1.04) | (-1.24, 1.81) | (-1.93, -0.12) |
|  |  |  |  |  |  |  |  |  |  |  |  |  |
| Ed: > H.S. Grad/Equiv | 0.46 | 0.02 | -0.62 | 0.49 | 0.05 | -0.64 | 0.53 | 0.03 | -0.62 | 0.47 | 0.01 | -0.62 |
|  | (-0.88, 1.80) | (-1.68, 1.71) | (-1.68, 0.44) | (-0.85, 1.83) | (-1.65, 1.75) | (-1.70, 0.42) | (-0.80, 1.87) | (-1.66, 1.72) | (-1.68, 0.44) | (-0.86, 1.81) | (-1.68, 1.70) | (-1.68, 0.44) |
|  |  |  |  |  |  |  |  |  |  |  |  |  |
| Blk. Grp. Poverty % | 0.00 | 0.01 | 0.01 | 0.00 | 0.01 | 0.01 | 0.00 | 0.01 | 0.01 | 0.00 | 0.01 | 0.01 |
|  | (-0.03, 0.04) | (-0.03, 0.06) | (-0.02, 0.04) | (-0.03, 0.04) | (-0.03, 0.06) | (-0.02, 0.04) | (-0.03, 0.04) | (-0.03, 0.06) | (-0.02, 0.04) | (-0.03, 0.04) | (-0.03, 0.06) | (-0.02, 0.04) |
|  |  |  |  |  |  |  |  |  |  |  |  |  |
| Platform: 850K | -0.54 | -0.24 | -0.02 | -0.51 | -0.24 | -0.01 | -0.50 | -0.27 | 0.01 | -0.52 | -0.32 | 0.02 |
|  | (-1.60, 0.52) | (-1.49, 1.00) | (-0.73, 0.70) | (-1.56, 0.55) | (-1.48, 1.00) | (-0.72, 0.70) | (-1.55, 0.55) | (-1.51, 0.97) | (-0.70, 0.72) | (-1.58, 0.53) | (-1.56, 0.92) | (-0.69, 0.73) |
|  |  |  |  |  |  |  |  |  |  |  |  |  |
| Constant | -0.11 | -3.10 | -3.04^*^ | 0.05 | -3.01 | -3.06^*^ | 0.12 | -3.12 | -2.98^*^ | -0.05 | -3.41 | -2.94^*^ |
|  | (-3.29, 3.08) | (-7.17, 0.98) | (-5.63, -0.44) | (-3.15, 3.25) | (-7.10, 1.08) | (-5.66, -0.46) | (-3.06, 3.31) | (-7.19, 0.94) | (-5.57, -0.38) | (-3.25, 3.15) | (-7.49, 0.67) | (-5.54, -0.34) |
|  |  |  |  |  |  |  |  |  |  |  |  |  |
|  | | | | | | | | | | | | |
| Observations | 478 | 478 | 478 | 478 | 478 | 478 | 478 | 478 | 478 | 478 | 478 | 478 |
|  | | | | | | | | | | | | |
| Note: | ^*^p<0.05, ^**^p<0.01, ^***^p<0.001 | | | | | | | | | | | |

**Supplemental Table 9.** Associations between linear natural-log transformed serum Pb and Hg and upper tertile (versus middle or lower tertile) serum Pb and Hg and accelerated Horvath Age, PhenoAge, and GrimAge among participants with DNAm measured on the Illumina 450K platform. All models include participant random effects and control for participant sex, race, ethnicity, cigarette use, income, education, and block group poverty.

|  | | | | | | | | | | | | |
| --- | --- | --- | --- | --- | --- | --- | --- | --- | --- | --- | --- | --- |
|  | Dependent variable: | | | | | | | | | | | |
|  |  | | | | | | | | | | | |
|  | Horvath Age | Pheno Age | Grim Age | Horvath Age | Pheno Age | Grim Age | Horvath Age | Pheno Age | Grim Age | Horvath Age | Pheno Age | Grim Age |
|  | (1) | (2) | (3) | (4) | (5) | (6) | (7) | (8) | (9) | (10) | (11) | (12) |
|  | | | | | | | | | | | | |
| Linear Serum ln(Pb) | -0.37 | -0.50 | -0.01 |  |  |  |  |  |  |  |  |  |
|  | (-1.39, 0.65) | (-1.71, 0.71) | (-0.76, 0.75) |  |  |  |  |  |  |  |  |  |
|  |  |  |  |  |  |  |  |  |  |  |  |  |
| Elevated Serum Pb (highest tertile) |  |  |  | 0.01 | -0.64 | 0.03 |  |  |  |  |  |  |
|  |  |  |  | (-2.33, 2.35) | (-3.42, 2.14) | (-1.70, 1.76) |  |  |  |  |  |  |
|  |  |  |  |  |  |  |  |  |  |  |  |  |
| Linear Serum ln(Hg) |  |  |  |  |  |  | -0.37 | 0.16 | 0.31 |  |  |  |
|  |  |  |  |  |  |  | (-1.34, 0.60) | (-1.00, 1.31) | (-0.40, 1.03) |  |  |  |
|  |  |  |  |  |  |  |  |  |  |  |  |  |
| Elevated Serum Hg (highest tertile) |  |  |  |  |  |  |  |  |  | -0.54 | 0.03 | -0.48 |
|  |  |  |  |  |  |  |  |  |  | (-2.85, 1.77) | (-2.72, 2.79) | (-2.19, 1.22) |
|  |  |  |  |  |  |  |  |  |  |  |  |  |
| Female | -0.22 | 2.24 | -1.18 | -0.11 | 2.31 | -1.17 | -0.10 | 2.35 | -1.20 | -0.09 | 2.36 | -1.11 |
|  | (-2.54, 2.09) | (-0.51, 4.99) | (-2.89, 0.53) | (-2.43, 2.21) | (-0.44, 5.06) | (-2.89, 0.54) | (-2.44, 2.25) | (-0.45, 5.14) | (-2.93, 0.53) | (-2.45, 2.26) | (-0.44, 5.17) | (-2.85, 0.62) |
|  |  |  |  |  |  |  |  |  |  |  |  |  |
| Race/Eth: non-Hisp. Afr. Am. | 0.48 | 1.51 | 1.70 | 0.53 | 1.50 | 1.70 | 0.29 | 1.51 | 1.81 | 0.38 | 1.46 | 1.70 |
|  | (-2.71, 3.67) | (-2.28, 5.29) | (-0.67, 4.06) | (-2.68, 3.73) | (-2.30, 5.30) | (-0.67, 4.07) | (-3.00, 3.58) | (-2.42, 5.44) | (-0.62, 4.25) | (-2.91, 3.66) | (-2.45, 5.38) | (-0.73, 4.12) |
|  |  |  |  |  |  |  |  |  |  |  |  |  |
| Race/Eth: Other | 0.59 | 1.73 | -0.60 | 0.69 | 1.60 | -0.59 | 0.46 | 1.85 | -0.51 | 0.42 | 1.78 | -0.88 |
|  | (-6.87, 8.06) | (-7.13, 10.59) | (-6.13, 4.92) | (-6.84, 8.23) | (-7.34, 10.54) | (-6.16, 4.98) | (-7.09, 8.00) | (-7.16, 10.85) | (-6.08, 5.07) | (-7.19, 8.02) | (-7.28, 10.85) | (-6.50, 4.74) |
|  |  |  |  |  |  |  |  |  |  |  |  |  |
| Ever Smoker | 2.04 | 2.03 | 4.47^***^ | 2.03 | 2.07 | 4.46^***^ | 2.33 | 1.89 | 4.21^***^ | 2.23 | 1.94 | 4.31^***^ |
|  | (-0.54, 4.62) | (-1.03, 5.09) | (2.56, 6.38) | (-0.56, 4.62) | (-1.00, 5.15) | (2.55, 6.38) | (-0.34, 5.00) | (-1.29, 5.08) | (2.23, 6.18) | (-0.43, 4.89) | (-1.23, 5.11) | (2.34, 6.27) |
|  |  |  |  |  |  |  |  |  |  |  |  |  |
| Income: >= 25k | 0.46 | 1.44 | -1.05 | 0.51 | 1.53 | -1.05 | 0.29 | 1.38 | -0.99 | 0.27 | 1.37 | -1.08 |
|  | (-1.96, 2.89) | (-1.44, 4.31) | (-2.85, 0.74) | (-1.91, 2.94) | (-1.35, 4.41) | (-2.84, 0.74) | (-2.18, 2.75) | (-1.56, 4.33) | (-2.82, 0.83) | (-2.21, 2.75) | (-1.58, 4.33) | (-2.91, 0.75) |
|  |  |  |  |  |  |  |  |  |  |  |  |  |
| Ed: > H.S. Grad/Equiv | 0.73 | -1.08 | -1.18 | 0.68 | -1.12 | -1.18 | 0.64 | -0.98 | -1.01 | 0.72 | -1.02 | -1.12 |
|  | (-1.68, 3.15) | (-3.95, 1.78) | (-2.96, 0.61) | (-1.73, 3.10) | (-3.98, 1.75) | (-2.96, 0.60) | (-1.82, 3.10) | (-3.91, 1.95) | (-2.83, 0.81) | (-1.73, 3.17) | (-3.94, 1.90) | (-2.93, 0.69) |
|  |  |  |  |  |  |  |  |  |  |  |  |  |
| Blk. Grp. Poverty % | 0.00 | 0.01 | -0.02 | 0.01 | 0.01 | -0.02 | 0.01 | 0.02 | -0.03 | 0.01 | 0.02 | -0.02 |
|  | (-0.06, 0.07) | (-0.07, 0.09) | (-0.07, 0.03) | (-0.06, 0.07) | (-0.06, 0.09) | (-0.07, 0.03) | (-0.05, 0.08) | (-0.06, 0.10) | (-0.08, 0.02) | (-0.05, 0.08) | (-0.06, 0.10) | (-0.07, 0.03) |
|  |  |  |  |  |  |  |  |  |  |  |  |  |
| Constant | -3.06 | -5.43 | -2.25 | -2.77 | -4.81 | -2.25 | -2.94 | -5.04 | -1.95 | -2.77 | -5.04 | -1.76 |
|  | (-8.20, 2.07) | (-11.53, 0.66) | (-6.04, 1.55) | (-7.91, 2.37) | (-10.91, 1.29) | (-6.05, 1.55) | (-8.10, 2.22) | (-11.19, 1.12) | (-5.76, 1.86) | (-7.99, 2.46) | (-11.28, 1.19) | (-5.63, 2.10) |
|  |  |  |  |  |  |  |  |  |  |  |  |  |
|  | | | | | | | | | | | | |
| Observations | 164 | 164 | 164 | 164 | 164 | 164 | 160 | 160 | 160 | 160 | 160 | 160 |
|  | | | | | | | | | | | | |
| Note: | ^*^p<0.05, ^**^p<0.01, ^***^p<0.001 | | | | | | | | | | | |

**Supplemental Table 10.** Associations between linear natural-log transformed serum Mn and Cu and upper/lower 15^th^ percentile (versus 16^th^-84^th^ percentile) serum Mn and Cu and accelerated Horvath Age, PhenoAge, and GrimAge among participants with DNAm measured on the Illumina 450K platform. All models include participant random effects and control for participant sex, race, ethnicity, cigarette use, income, education, and block group poverty.

|  | | | | | | | | | | | | |
| --- | --- | --- | --- | --- | --- | --- | --- | --- | --- | --- | --- | --- |
|  | Dependent variable: | | | | | | | | | | | |
|  |  | | | | | | | | | | | |
|  | Horvath Age | Pheno Age | Grim Age | Horvath Age | Pheno Age | Grim Age | Horvath Age | Pheno Age | Grim Age | Horvath Age | Pheno Age | Grim Age |
|  | (1) | (2) | (3) | (4) | (5) | (6) | (7) | (8) | (9) | (10) | (11) | (12) |
|  | | | | | | | | | | | | |
| Linear Serum ln(Mn) | -0.46 | -1.01 | -0.44 |  |  |  |  |  |  |  |  |  |
|  | (-1.94, 1.03) | (-2.77, 0.74) | (-1.54, 0.65) |  |  |  |  |  |  |  |  |  |
|  |  |  |  |  |  |  |  |  |  |  |  |  |
| Abnormal Serum Mn (highest/lowest 15%tile) |  |  |  | 0.42 | -0.98 | 0.55 |  |  |  |  |  |  |
|  |  |  |  | (-2.00, 2.83) | (-3.84, 1.88) | (-1.23, 2.33) |  |  |  |  |  |  |
|  |  |  |  |  |  |  |  |  |  |  |  |  |
| Linear Serum ln(Cu) |  |  |  |  |  |  | 0.02 | 2.37 | 0.52 |  |  |  |
|  |  |  |  |  |  |  | (-2.81, 2.84) | (-0.97, 5.71) | (-1.57, 2.61) |  |  |  |
|  |  |  |  |  |  |  |  |  |  |  |  |  |
| Abnormal Serum Cu (highest/lowest 15%tile) |  |  |  |  |  |  |  |  |  | -0.31 | -0.18 | -1.00 |
|  |  |  |  |  |  |  |  |  |  | (-2.85, 2.22) | (-3.19, 2.83) | (-2.86, 0.87) |
|  |  |  |  |  |  |  |  |  |  |  |  |  |
| Female | -0.10 | 2.43 | -1.16 | -0.14 | 2.45 | -1.21 | -0.11 | 1.95 | -1.27 | -0.10 | 2.40 | -1.14 |
|  | (-2.39, 2.20) | (-0.29, 5.15) | (-2.86, 0.53) | (-2.44, 2.17) | (-0.28, 5.18) | (-2.91, 0.49) | (-2.47, 2.24) | (-0.83, 4.74) | (-3.01, 0.47) | (-2.40, 2.20) | (-0.33, 5.13) | (-2.84, 0.55) |
|  |  |  |  |  |  |  |  |  |  |  |  |  |
| Race/Eth: non-Hisp. Afr. Am. | 0.44 | 1.36 | 1.61 | 0.59 | 1.42 | 1.78 | 0.52 | 1.16 | 1.61 | 0.51 | 1.55 | 1.65 |
|  | (-2.77, 3.64) | (-2.43, 5.16) | (-0.75, 3.98) | (-2.63, 3.80) | (-2.39, 5.23) | (-0.59, 4.15) | (-2.71, 3.75) | (-2.65, 4.98) | (-0.78, 3.99) | (-2.68, 3.71) | (-2.24, 5.35) | (-0.70, 4.01) |
|  |  |  |  |  |  |  |  |  |  |  |  |  |
| Race/Eth: Other | 0.56 | 1.58 | -0.72 | 0.87 | 1.45 | -0.36 | 0.69 | 1.99 | -0.57 | 0.68 | 1.86 | -0.63 |
|  | (-6.91, 8.04) | (-7.28, 10.43) | (-6.24, 4.80) | (-6.67, 8.40) | (-7.50, 10.39) | (-5.93, 5.20) | (-6.78, 8.16) | (-6.83, 10.81) | (-6.09, 4.95) | (-6.79, 8.15) | (-7.02, 10.73) | (-6.14, 4.87) |
|  |  |  |  |  |  |  |  |  |  |  |  |  |
| Ever Smoker | 2.11 | 2.19 | 4.54^***^ | 2.00 | 2.10 | 4.42^***^ | 2.03 | 1.78 | 4.41^***^ | 2.05 | 2.03 | 4.51^***^ |
|  | (-0.48, 4.70) | (-0.88, 5.27) | (2.63, 6.46) | (-0.59, 4.59) | (-0.97, 5.17) | (2.51, 6.33) | (-0.57, 4.63) | (-1.29, 4.85) | (2.49, 6.33) | (-0.54, 4.63) | (-1.05, 5.10) | (2.61, 6.42) |
|  |  |  |  |  |  |  |  |  |  |  |  |  |
| Income: >= 25k | 0.56 | 1.60 | -1.01 | 0.54 | 1.44 | -1.01 | 0.52 | 1.71 | -1.01 | 0.53 | 1.52 | -0.99 |
|  | (-1.87, 2.98) | (-1.27, 4.47) | (-2.80, 0.78) | (-1.88, 2.97) | (-1.44, 4.32) | (-2.81, 0.78) | (-1.92, 2.95) | (-1.17, 4.58) | (-2.80, 0.79) | (-1.89, 2.96) | (-1.37, 4.40) | (-2.78, 0.80) |
|  |  |  |  |  |  |  |  |  |  |  |  |  |
| Ed: > H.S. Grad/Equiv | 0.75 | -1.01 | -1.12 | 0.64 | -1.04 | -1.24 | 0.68 | -1.24 | -1.20 | 0.70 | -1.14 | -1.13 |
|  | (-1.67, 3.17) | (-3.87, 1.86) | (-2.90, 0.67) | (-1.79, 3.06) | (-3.92, 1.84) | (-3.03, 0.55) | (-1.73, 3.10) | (-4.09, 1.61) | (-2.98, 0.58) | (-1.72, 3.11) | (-4.01, 1.73) | (-2.91, 0.65) |
|  |  |  |  |  |  |  |  |  |  |  |  |  |
| Blk. Grp. Poverty % | 0.01 | 0.01 | -0.02 | 0.01 | 0.02 | -0.02 | 0.01 | 0.02 | -0.02 | 0.01 | 0.01 | -0.02 |
|  | (-0.06, 0.07) | (-0.07, 0.09) | (-0.07, 0.03) | (-0.06, 0.07) | (-0.06, 0.09) | (-0.07, 0.03) | (-0.06, 0.07) | (-0.06, 0.10) | (-0.07, 0.03) | (-0.06, 0.07) | (-0.06, 0.09) | (-0.07, 0.03) |
|  |  |  |  |  |  |  |  |  |  |  |  |  |
| Constant | -2.59 | -4.63 | -2.07 | -2.85 | -4.84 | -2.35 | -2.87 | -21.55 | -5.88 | -2.75 | -5.02 | -2.18 |
|  | (-7.69, 2.52) | (-10.68, 1.41) | (-5.83, 1.70) | (-7.94, 2.25) | (-10.89, 1.20) | (-6.11, 1.41) | (-23.22, 17.47) | (-45.57, 2.47) | (-20.91, 9.14) | (-7.82, 2.33) | (-11.05, 1.01) | (-5.92, 1.56) |
|  |  |  |  |  |  |  |  |  |  |  |  |  |
|  | | | | | | | | | | | | |
| Observations | 164 | 164 | 164 | 164 | 164 | 164 | 164 | 164 | 164 | 164 | 164 | 164 |
|  | | | | | | | | | | | | |
| Note: | ^*^p<0.05, ^**^p<0.01, ^***^p<0.001 | | | | | | | | | | | |

**Supplemental Table 11.** Associations between linear natural-log transformed serum Pb and Hg and upper tertile (versus middle or lower tertile) serum Pb and Hg and accelerated Horvath Age, PhenoAge, and GrimAge among participants with DNAm measured on the Illumina 850K platform. All models include participant random effects and control for participant sex, race, ethnicity, cigarette use, income, education, and block group poverty.

|  | | | | | | | | | | | | |
| --- | --- | --- | --- | --- | --- | --- | --- | --- | --- | --- | --- | --- |
|  | Dependent variable: | | | | | | | | | | | |
|  |  | | | | | | | | | | | |
|  | Horvath Age | Pheno Age | Grim Age | Horvath Age | Pheno Age | Grim Age | Horvath Age | Pheno Age | Grim Age | Horvath Age | Pheno Age | Grim Age |
|  | (1) | (2) | (3) | (4) | (5) | (6) | (7) | (8) | (9) | (10) | (11) | (12) |
|  | | | | | | | | | | | | |
| Linear Serum ln(Pb) | 0.08 | 0.18 | 0.18 |  |  |  |  |  |  |  |  |  |
|  | (-0.38, 0.54) | (-0.35, 0.71) | (-0.14, 0.49) |  |  |  |  |  |  |  |  |  |
|  |  |  |  |  |  |  |  |  |  |  |  |  |
| Elevated Serum Pb (highest tertile) |  |  |  | -0.56 | 0.28 | 0.50 |  |  |  |  |  |  |
|  |  |  |  | (-1.60, 0.48) | (-0.90, 1.47) | (-0.20, 1.20) |  |  |  |  |  |  |
|  |  |  |  |  |  |  |  |  |  |  |  |  |
| Linear Serum ln(Hg) |  |  |  |  |  |  | -0.10 | 0.10 | -0.04 |  |  |  |
|  |  |  |  |  |  |  | (-0.48, 0.28) | (-0.35, 0.54) | (-0.31, 0.22) |  |  |  |
|  |  |  |  |  |  |  |  |  |  |  |  |  |
| Elevated Serum Hg (highest tertile) |  |  |  |  |  |  |  |  |  | -0.68 | -0.19 | -0.05 |
|  |  |  |  |  |  |  |  |  |  | (-1.77, 0.40) | (-1.44, 1.07) | (-0.80, 0.69) |
|  |  |  |  |  |  |  |  |  |  |  |  |  |
| Female | -0.30 | -1.70 | -2.63^***^ | -0.32 | -1.68 | -2.61^***^ | -0.16 | -1.60 | -2.53^***^ | -0.17 | -1.59 | -2.53^***^ |
|  | (-1.81, 1.21) | (-3.89, 0.49) | (-4.07, -1.19) | (-1.83, 1.18) | (-3.87, 0.50) | (-4.05, -1.17) | (-1.67, 1.36) | (-3.82, 0.63) | (-3.99, -1.07) | (-1.68, 1.34) | (-3.82, 0.63) | (-3.99, -1.07) |
|  |  |  |  |  |  |  |  |  |  |  |  |  |
| Race/Eth: non-Hisp. Afr. Am. | -2.97 | -0.58 | 0.49 | -2.88 | -0.59 | 0.46 | -2.90 | -0.57 | 0.57 | -2.89 | -0.58 | 0.57 |
|  | (-6.66, 0.73) | (-6.08, 4.91) | (-3.14, 4.13) | (-6.55, 0.79) | (-6.07, 4.90) | (-3.17, 4.09) | (-6.58, 0.77) | (-6.10, 4.97) | (-3.09, 4.23) | (-6.56, 0.77) | (-6.11, 4.95) | (-3.08, 4.23) |
|  |  |  |  |  |  |  |  |  |  |  |  |  |
| Race/Eth: Other | -4.20 | -1.07 | 0.22 | -4.05 | -1.12 | 0.12 | -4.21 | -1.15 | 0.20 | -3.99 | -1.09 | 0.22 |
|  | (-10.69, 2.29) | (-10.63, 8.49) | (-6.09, 6.53) | (-10.51, 2.41) | (-10.68, 8.43) | (-6.18, 6.42) | (-10.68, 2.26) | (-10.79, 8.49) | (-6.15, 6.55) | (-10.45, 2.48) | (-10.74, 8.55) | (-6.13, 6.57) |
|  |  |  |  |  |  |  |  |  |  |  |  |  |
| Ever Smoker | -0.42 | 1.74 | 4.50^***^ | -0.38 | 1.75 | 4.50^***^ | -0.36 | 1.73 | 4.59^***^ | -0.29 | 1.81 | 4.58^***^ |
|  | (-2.12, 1.29) | (-0.73, 4.21) | (2.88, 6.12) | (-2.08, 1.31) | (-0.72, 4.22) | (2.88, 6.12) | (-2.08, 1.36) | (-0.78, 4.25) | (2.95, 6.23) | (-2.01, 1.42) | (-0.70, 4.32) | (2.94, 6.22) |
|  |  |  |  |  |  |  |  |  |  |  |  |  |
| Income: >= 25k | -0.53 | -0.09 | -0.74 | -0.56 | -0.06 | -0.69 | -0.77 | 0.00 | -0.64 | -0.70 | 0.05 | -0.64 |
|  | (-1.89, 0.83) | (-1.79, 1.61) | (-1.77, 0.30) | (-1.91, 0.80) | (-1.76, 1.65) | (-1.72, 0.35) | (-2.14, 0.60) | (-1.74, 1.75) | (-1.71, 0.43) | (-2.08, 0.67) | (-1.71, 1.80) | (-1.71, 0.43) |
|  |  |  |  |  |  |  |  |  |  |  |  |  |
| Ed: > H.S. Grad/Equiv | -0.00 | -0.18 | -0.73 | 0.02 | -0.18 | -0.73 | -0.05 | -0.03 | -0.80 | -0.12 | -0.10 | -0.79 |
|  | (-1.45, 1.44) | (-2.15, 1.80) | (-1.98, 0.53) | (-1.42, 1.46) | (-2.15, 1.80) | (-1.98, 0.53) | (-1.51, 1.42) | (-2.08, 2.02) | (-2.10, 0.51) | (-1.58, 1.35) | (-2.16, 1.95) | (-2.11, 0.52) |
|  |  |  |  |  |  |  |  |  |  |  |  |  |
| Blk. Grp. Poverty % | -0.01 | 0.01 | 0.02 | -0.01 | 0.01 | 0.02 | -0.01 | 0.01 | 0.02 | -0.01 | 0.01 | 0.02 |
|  | (-0.04, 0.03) | (-0.04, 0.06) | (-0.01, 0.05) | (-0.05, 0.03) | (-0.04, 0.06) | (-0.01, 0.05) | (-0.05, 0.03) | (-0.04, 0.06) | (-0.01, 0.06) | (-0.04, 0.03) | (-0.04, 0.06) | (-0.01, 0.06) |
|  |  |  |  |  |  |  |  |  |  |  |  |  |
| Constant | 3.89 | 0.41 | -2.09 | 3.93 | 0.06 | -2.51 | 3.76 | -0.01 | -2.53 | 3.90 | -0.08 | -2.49 |
|  | (-0.39, 8.17) | (-5.82, 6.64) | (-6.19, 2.01) | (-0.28, 8.14) | (-6.13, 6.25) | (-6.58, 1.56) | (-0.48, 8.01) | (-6.29, 6.27) | (-6.65, 1.59) | (-0.32, 8.13) | (-6.35, 6.18) | (-6.60, 1.63) |
|  |  |  |  |  |  |  |  |  |  |  |  |  |
|  | | | | | | | | | | | | |
| Observations | 314 | 314 | 314 | 314 | 314 | 314 | 307 | 307 | 307 | 307 | 307 | 307 |
|  | | | | | | | | | | | | |
| Note: | ^*^p<0.05, ^**^p<0.01, ^***^p<0.001 | | | | | | | | | | | |

**Supplemental Table 12.** Associations between linear natural-log transformed serum Mn and Cu and upper/lower 15^th^ percentile (versus 16^th^-84^th^ percentile) serum Mn and Cu and accelerated Horvath Age, PhenoAge, and GrimAge among participants with DNAm measured on the Illumina 850K platform. All models include participant random effects and control for participant sex, race, ethnicity, cigarette use, income, education, and block group poverty.

|  | | | | | | | | | | | | |
| --- | --- | --- | --- | --- | --- | --- | --- | --- | --- | --- | --- | --- |
|  | Dependent variable: | | | | | | | | | | | |
|  |  | | | | | | | | | | | |
|  | Horvath Age | Pheno Age | Grim Age | Horvath Age | Pheno Age | Grim Age | Horvath Age | Pheno Age | Grim Age | Horvath Age | Pheno Age | Grim Age |
|  | (1) | (2) | (3) | (4) | (5) | (6) | (7) | (8) | (9) | (10) | (11) | (12) |
|  | | | | | | | | | | | | |
| Linear Serum ln(Mn) | 0.01 | 0.64 | 0.28 |  |  |  |  |  |  |  |  |  |
|  | (-0.84, 0.86) | (-0.36, 1.64) | (-0.32, 0.88) |  |  |  |  |  |  |  |  |  |
|  |  |  |  |  |  |  |  |  |  |  |  |  |
| Abnormal Serum Mn (highest/lowest 15%tile) |  |  |  | -0.05 | -0.64 | 0.16 |  |  |  |  |  |  |
|  |  |  |  | (-1.13, 1.04) | (-1.88, 0.61) | (-0.58, 0.90) |  |  |  |  |  |  |
|  |  |  |  |  |  |  |  |  |  |  |  |  |
| Linear Serum ln(Cu) |  |  |  |  |  |  | -0.15 | -0.29 | -0.26 |  |  |  |
|  |  |  |  |  |  |  | (-0.95, 0.65) | (-1.21, 0.62) | (-0.80, 0.28) |  |  |  |
|  |  |  |  |  |  |  |  |  |  |  |  |  |
| Abnormal Serum Cu (highest/lowest 15%tile) |  |  |  |  |  |  |  |  |  | -0.27 | 0.48 | 0.03 |
|  |  |  |  |  |  |  |  |  |  | (-1.35, 0.81) | (-0.75, 1.71) | (-0.70, 0.75) |
|  |  |  |  |  |  |  |  |  |  |  |  |  |
| Female | -0.30 | -1.76 | -2.65^***^ | -0.30 | -1.70 | -2.62^***^ | -0.29 | -1.67 | -2.60^***^ | -0.33 | -1.65 | -2.62^***^ |
|  | (-1.82, 1.21) | (-3.95, 0.43) | (-4.09, -1.21) | (-1.81, 1.21) | (-3.89, 0.49) | (-4.06, -1.18) | (-1.80, 1.22) | (-3.85, 0.52) | (-4.04, -1.16) | (-1.84, 1.19) | (-3.84, 0.54) | (-4.06, -1.18) |
|  |  |  |  |  |  |  |  |  |  |  |  |  |
| Race/Eth: non-Hisp. Afr. Am. | -2.95 | -0.60 | 0.51 | -2.94 | -0.47 | 0.51 | -2.93 | -0.50 | 0.57 | -3.01 | -0.45 | 0.54 |
|  | (-6.64, 0.74) | (-6.09, 4.89) | (-3.13, 4.15) | (-6.64, 0.75) | (-5.97, 5.03) | (-3.13, 4.15) | (-6.62, 0.77) | (-5.98, 4.98) | (-3.07, 4.22) | (-6.70, 0.69) | (-5.93, 5.03) | (-3.11, 4.18) |
|  |  |  |  |  |  |  |  |  |  |  |  |  |
| Race/Eth: Other | -4.19 | -1.00 | 0.26 | -4.19 | -0.94 | 0.20 | -4.16 | -1.00 | 0.29 | -4.29 | -0.88 | 0.24 |
|  | (-10.68, 2.30) | (-10.56, 8.56) | (-6.05, 6.58) | (-10.68, 2.31) | (-10.51, 8.63) | (-6.12, 6.52) | (-10.66, 2.35) | (-10.54, 8.55) | (-6.03, 6.61) | (-10.80, 2.22) | (-10.43, 8.66) | (-6.08, 6.56) |
|  |  |  |  |  |  |  |  |  |  |  |  |  |
| Ever Smoker | -0.41 | 1.84 | 4.55^***^ | -0.41 | 1.77 | 4.52^***^ | -0.41 | 1.75 | 4.51^***^ | -0.40 | 1.75 | 4.52^***^ |
|  | (-2.11, 1.30) | (-0.64, 4.31) | (2.93, 6.18) | (-2.11, 1.30) | (-0.71, 4.24) | (2.90, 6.14) | (-2.12, 1.30) | (-0.71, 4.22) | (2.89, 6.14) | (-2.11, 1.31) | (-0.71, 4.21) | (2.90, 6.14) |
|  |  |  |  |  |  |  |  |  |  |  |  |  |
| Income: >= 25k | -0.53 | -0.14 | -0.74 | -0.53 | -0.09 | -0.71 | -0.51 | -0.04 | -0.68 | -0.50 | -0.12 | -0.72 |
|  | (-1.89, 0.84) | (-1.84, 1.57) | (-1.79, 0.30) | (-1.89, 0.83) | (-1.80, 1.61) | (-1.76, 0.33) | (-1.87, 0.86) | (-1.74, 1.67) | (-1.72, 0.36) | (-1.87, 0.86) | (-1.83, 1.58) | (-1.77, 0.32) |
|  |  |  |  |  |  |  |  |  |  |  |  |  |
| Ed: > H.S. Grad/Equiv | 0.00 | -0.22 | -0.74 | 0.00 | -0.17 | -0.71 | -0.01 | -0.19 | -0.73 | 0.00 | -0.18 | -0.71 |
|  | (-1.44, 1.45) | (-2.20, 1.75) | (-2.00, 0.52) | (-1.44, 1.45) | (-2.15, 1.81) | (-1.96, 0.55) | (-1.45, 1.44) | (-2.16, 1.79) | (-1.99, 0.53) | (-1.44, 1.45) | (-2.15, 1.79) | (-1.97, 0.55) |
|  |  |  |  |  |  |  |  |  |  |  |  |  |
| Blk. Grp. Poverty % | -0.01 | 0.01 | 0.02 | -0.01 | 0.01 | 0.02 | -0.01 | 0.01 | 0.02 | -0.01 | 0.01 | 0.02 |
|  | (-0.04, 0.03) | (-0.04, 0.06) | (-0.01, 0.05) | (-0.04, 0.03) | (-0.05, 0.06) | (-0.01, 0.05) | (-0.04, 0.03) | (-0.05, 0.06) | (-0.01, 0.05) | (-0.04, 0.03) | (-0.05, 0.06) | (-0.01, 0.05) |
|  |  |  |  |  |  |  |  |  |  |  |  |  |
| Constant | 3.77 | -0.05 | -2.45 | 3.79 | 0.33 | -2.41 | 4.82 | 2.16 | -0.56 | 3.91 | -0.11 | -2.38 |
|  | (-0.46, 8.00) | (-6.24, 6.15) | (-6.52, 1.63) | (-0.45, 8.02) | (-5.87, 6.53) | (-6.49, 1.67) | (-2.11, 11.74) | (-6.66, 10.98) | (-6.08, 4.96) | (-0.35, 8.17) | (-6.31, 6.09) | (-6.47, 1.71) |
|  |  |  |  |  |  |  |  |  |  |  |  |  |
|  | | | | | | | | | | | | |
| Observations | 314 | 314 | 314 | 314 | 314 | 314 | 314 | 314 | 314 | 314 | 314 | 314 |
|  | | | | | | | | | | | | |
| Note: | ^*^p<0.05, ^**^p<0.01, ^***^p<0.001 | | | | | | | | | | | |

**Supplemental Figure 5.** Associations between **(A)** linear natural-log transformed serum Pb, Hg, Mn, and Cu and **(B)** upper tertile (versus middle or lower tertile) serum Pb and Hg or upper/lower 15^th^ percentile (versus 16^th^-84^th^ percentile) serum Mn and Cu and Horvath Age, PhenoAge, and GrimAge. All models are restricted to Illumina 450K data only, include participant random effects, and control for participant sex, race, ethnicity, cigarette use, income, education, and block group poverty 95% C.I. = 95% Confidence Interval. Full model results are available in *Supplemental Tables 9-10*.


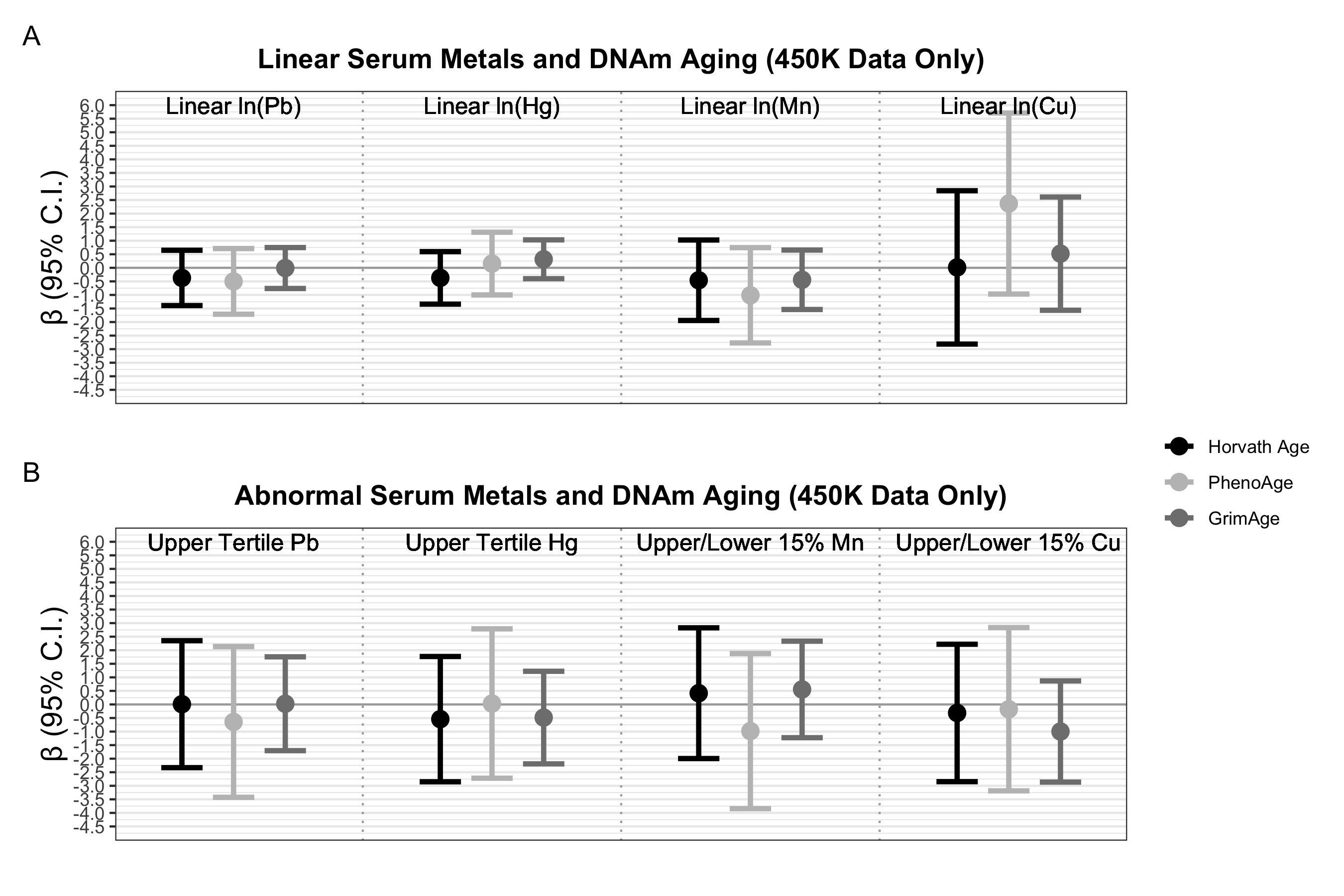


**Supplemental Figure 6.** Associations between **(A)** linear natural-log transformed serum Pb, Hg, Mn, and Cu and **(B)** upper tertile (versus middle or lower tertile) serum Pb and Hg or upper/lower 15^th^ percentile (versus 16^th^-84^th^ percentile) serum Mn and Cu and Horvath Age, PhenoAge, and GrimAge. All models are restricted to Illumina 850K data only, include participant random effects, and control for participant sex, race, ethnicity, cigarette use, income, education, and block group poverty. 95% C.I. = 95% Confidence Interval. Full model results are available in *Supplemental Tables 11-12*.


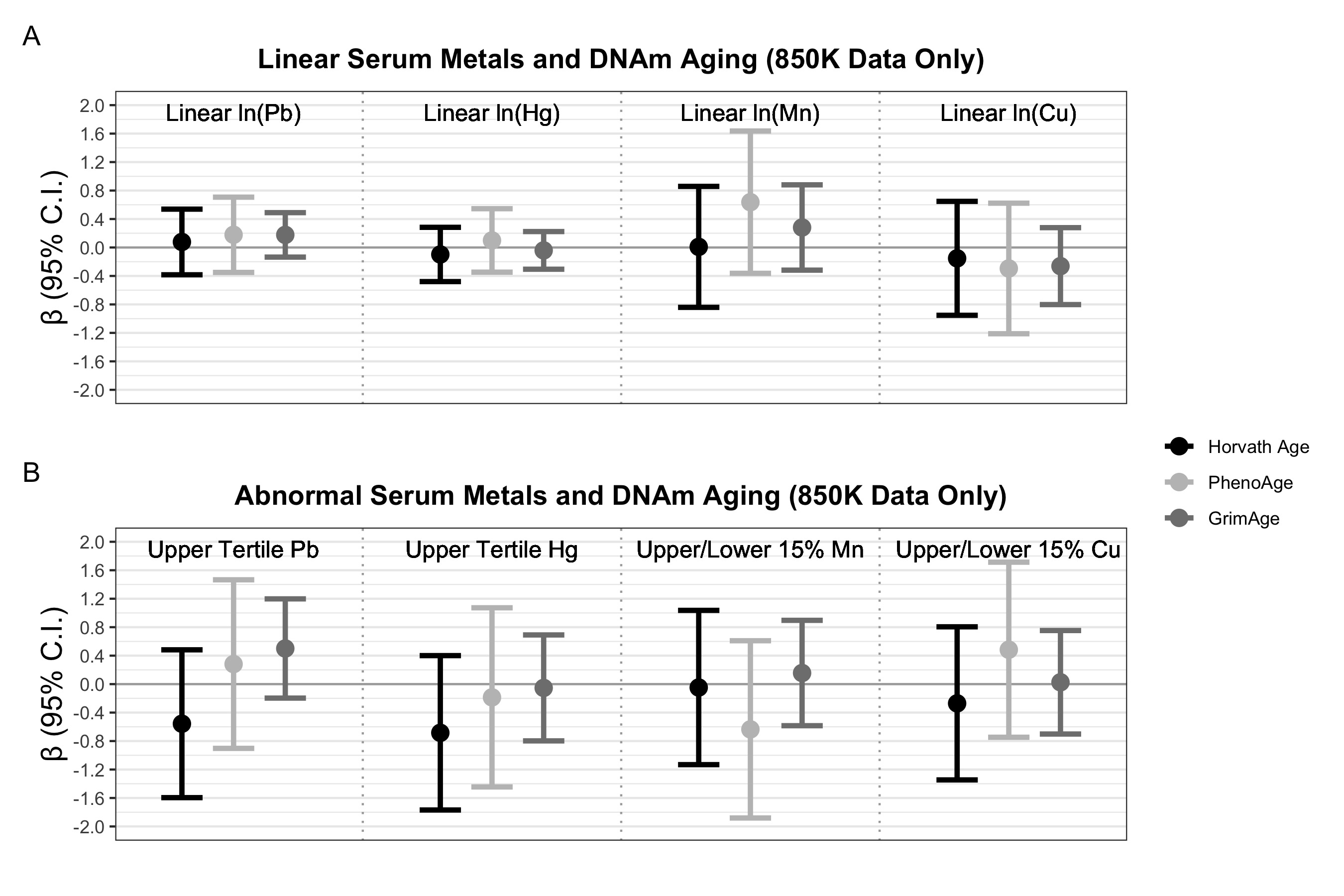


**Supplemental Table 13.** Total effect of metal mixture on Horvath Age, PhenoAge, and GrimAge estimated using BKMR. All models include participant random effects and control for participant sex, race, ethnicity, cigarette use, income, education, block group poverty, and Illumina platform. 95% B.C.I. = Bayesian credible interval.

|  | **Horvath Age** | | **PhenoAge** | | **GrimAge** | |
| --- | --- | --- | --- | --- | --- | --- |
| **Total Mixture Quantile** | **Estimate (β)** | **95% BCI** | **Estimate (β)** | **95% BCI** | **Estimate (β)** | **95% BCI** |
| **0.05** | -0.07 | -1.31 , 1.16 | -1.00 | -2.41 , 0.41 | -1.09 | -2.07 , -0.12 |
| **0.15** | 0.07 | -0.53 , 0.68 | -0.57 | -1.27 , 0.14 | -0.69 | -1.15 , -0.23 |
| **0.25** | 0.08 | -0.28 , 0.43 | -0.35 | -0.77 , 0.07 | -0.44 | -0.71 , -0.18 |
| **0.35** | 0.04 | -0.15 , 0.23 | -0.20 | -0.43 , 0.04 | -0.25 | -0.39 , -0.11 |
| **0.45** | 0.01 | -0.03 , 0.05 | -0.05 | -0.10 , 0.01 | -0.06 | -0.09 , -0.03 |
| **0.55** | -0.05 | -0.13 , 0.03 | 0.07 | -0.02 , 0.16 | 0.10 | 0.04 , 0.15 |
| **0.65** | -0.17 | -0.39 , 0.05 | 0.12 | -0.14 , 0.38 | 0.26 | 0.11 , 0.42 |
| **0.75** | -0.29 | -0.65 , 0.07 | 0.22 | -0.21 , 0.66 | 0.45 | 0.18 , 0.71 |
| **0.85** | -0.57 | -1.16 , 0.02 | 0.32 | -0.4 , 1.04 | 0.70 | 0.25 , 1.15 |
| **0.95** | -1.23 | -2.4 , -0.07 | 0.45 | -0.97 , 1.88 | 1.10 | 0.15 , 2.06 |

**Supplemental Table 14.** Estimated PIPs (posterior inclusion probabilities) for each metal and outcome. All models estimated using BKMR, include participant random effects, and control for participant sex, race, ethnicity, cigarette use, income, education, block group poverty, and Illumina platform.

| **Metal** | **Horvath Age** | **PhenoAge** | **GrimAge** |
| --- | --- | --- | --- |
| **ln(Pb)** | 0.18852 | 0.22308 | 0.31228 |
| **ln(Hg)** | 0.27416 | 0.17576 | 0.25460 |
| **ln(Mn)** | 0.29880 | 0.28364 | 0.36168 |
| **ln(Cu)** | 0.30736 | 0.43520 | 0.45112 |

**Supplemental Figure 7**. Estimated change in accelerated **(A)** Horvath Age, **(B)** PhenoAge, and **(C)** GrimAge after increasing a single metal from the 25^th^ to the 75^th^ percentile while holding all other metals constant at either the 25^th^, 50^th^, or 75^th^ percentile. All models estimated using BKMR, include participant random effects, and control for participant sex, race, ethnicity, cigarette use, income, education, block group poverty, and Illumina platform. 95% B.C.I. = Bayesian credible interval.


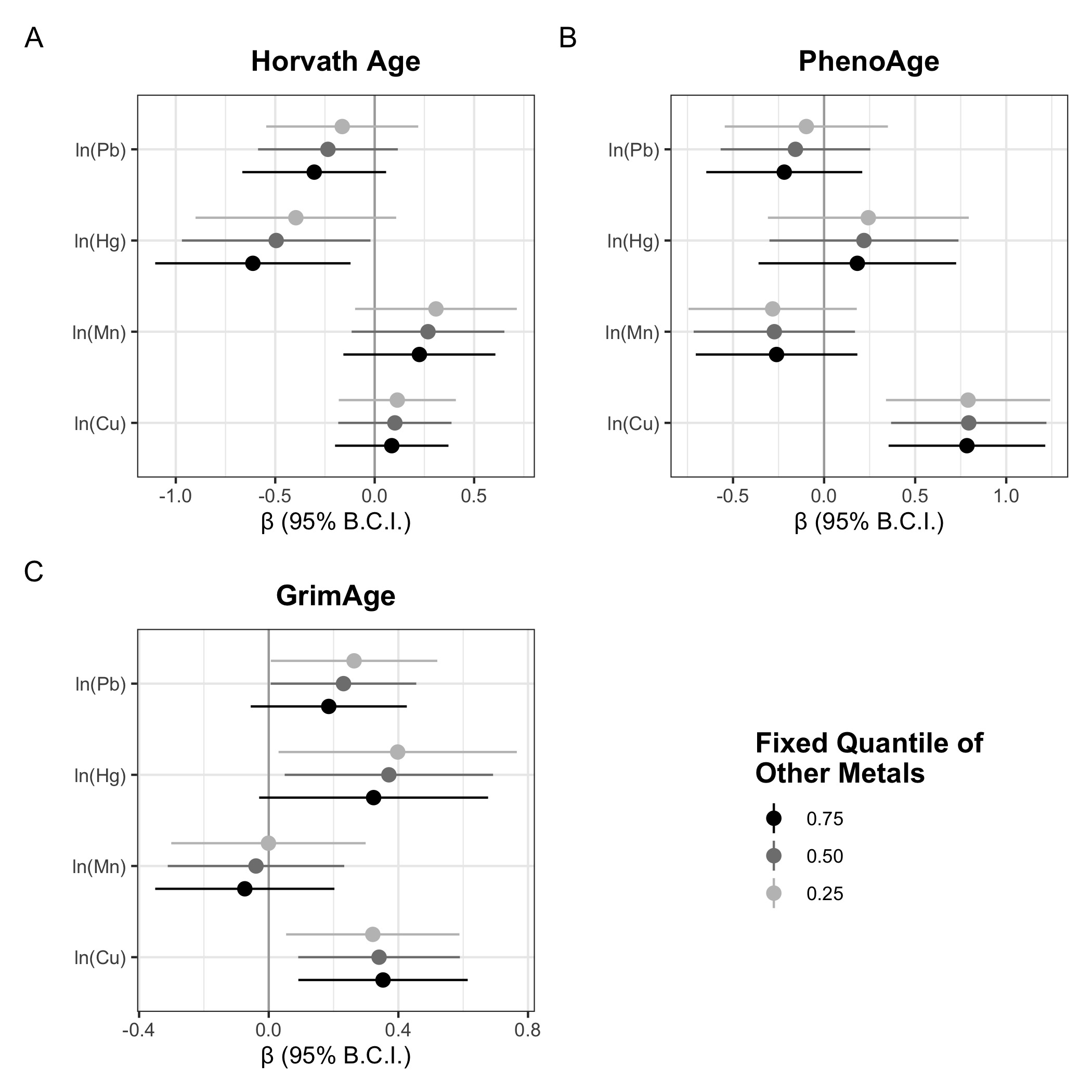


**Supplemental Table 15**. Estimated change in Horvath Age, PhenoAge, and GrimAge after increasing a single metal from the 25^th^ to the 75^th^ percentile while holding all other metals constant at either the 25^th^, 50^th^, or 75^th^ percentile. All models estimated using BKMR, include participant random effects, and control for participant gender, race, ethnicity, cigarette use, income, education, block group poverty, and Illumina platform. 95% B.C.I. = Bayesian credible interval.

|  |  | **other metals at 25^th^ percentile** | | **other metals at 50^th^ percentile** | | **other metals at 75^th^ percentile** | |
| --- | --- | --- | --- | --- | --- | --- | --- |
|  | **Metal** | **Estimate (β)** | **95% BCI** | **Estimate (β)** | **95% BCI** | **Estimate (β)** | **95% BCI** |
| **Horvath Age** | ln(Pb) | -0.16 | -0.55 , 0.22 | -0.24 | -0.59 , 0.12 | -0.3 | -0.67 , 0.06 |
|  | ln(Hg) | -0.4 | -0.9 , 0.11 | -0.5 | -0.97 , -0.02 | -0.61 | -1.1 , -0.12 |
|  | ln(Mn) | 0.31 | -0.1 , 0.71 | 0.27 | -0.12 , 0.65 | 0.22 | -0.16 , 0.61 |
|  | ln(Cu) | 0.11 | -0.18 , 0.41 | 0.1 | -0.18 , 0.39 | 0.09 | -0.2 , 0.37 |
| **PhenoAge** | ln(Pb) | -0.1 | -0.55 , 0.35 | -0.16 | -0.57 , 0.25 | -0.22 | -0.65 , 0.21 |
|  | ln(Hg) | 0.24 | -0.31 , 0.79 | 0.22 | -0.3 , 0.74 | 0.18 | -0.36 , 0.73 |
|  | ln(Mn) | -0.28 | -0.74 , 0.18 | -0.27 | -0.72 , 0.17 | -0.26 | -0.7 , 0.18 |
|  | ln(Cu) | 0.79 | 0.34 , 1.24 | 0.79 | 0.37 , 1.22 | 0.78 | 0.35 , 1.21 |
| **GrimAge** | ln(Pb) | 0.26 | 0.01 , 0.52 | 0.23 | 0.01 , 0.46 | 0.19 | -0.06 , 0.43 |
|  | ln(Hg) | 0.4 | 0.03 , 0.77 | 0.37 | 0.05 , 0.69 | 0.32 | -0.03 , 0.68 |
|  | ln(Mn) | 0 | -0.3 , 0.3 | -0.04 | -0.31 , 0.23 | -0.07 | -0.35 , 0.2 |
|  | ln(Cu) | 0.32 | 0.05 , 0.59 | 0.34 | 0.09 , 0.59 | 0.35 | 0.09 , 0.61 |
